# Global, regional, and national burden of NASH related liver cancer in adults aged 45 and above: an analysis from the GBD 2021 and forecast to 2050

**DOI:** 10.3389/fnut.2025.1651357

**Published:** 2025-08-19

**Authors:** Jianing Shi, Xuan Wu, Jiali Yu, Qingli Zhou

**Affiliations:** Department of Information Technology, The Fourth Affiliated Hospital of School of Medicine, and International School of Medicine, International Institutes of Medicine, Zhejiang University, Yiwu, China

**Keywords:** nonalcoholic steatohepatitis, liver cancer, global burden, prediction, prevalence

## Abstract

**Introduction:**

Nonalcoholic steatohepatitis (NASH) is a more advanced stage of Nonalcoholic fatty liver disease (NAFLD) and is particularly common among middle-aged and older adults. NASH-related liver cancer (NALC) is a serious consequence of NASH progression and has become one of the significant causes of global cancer mortality. However, there is currently no comprehensive analysis of the disease burden in this population. This study aims to comprehensively analyze the incidence of NALC among individuals aged 45 and above worldwide from 1990 to 2021 and to project the trends through 2050.

**Methods:**

Using the data from the Global Burden of Disease 2021, we analyzed incidence, prevalence, mortality, and disability-adjusted life years (DALYs) among adults ≥ 45 from 1990 to 2021, employing frontier risk analysis and Bayesian modeling to project trends through 2050.

**Results:**

In 2021, among adults aged 45 and above, the age-standardized incidence rate of NALC was 1.69 per 100,000, the prevalence rate was 2.01 per 100,000, the mortality rate was 1.66 per 100,000, and DALYs were 36.69 per 100,000 person-years. Since 1990, global indicators have steadily increased, with low-SDI regions showing the highest incidence, mortality, and DALYs, and high-SDI regions having the highest prevalence. Significant regional and national differences were observed. Future projections suggest that while incidence, mortality, and DALYs will initially rise and then decline, prevalence will continue to increase. By 2050, the age-standardized incidence rate of NALC was projected to reach 1.78 per 100,000, and its age-standardized prevalence rate was projected to reach 2.37 per 100,000 person-years. High fasting blood glucose and metabolic risks were the primary contributors to mortality.

**Conclusion:**

These findings suggested the urgent need for developing targeted strategies addressing metabolic-related risk factors and implementing standardized screening protocols to mitigate the growing disease burden of NALC in aging populations.

## 1 Introduction

For Nonalcoholic fatty liver disease (NAFLD) is a liver disease characterized by hepatic steatosis (HS), as demonstrated by imaging or histological evidence, after excluding other known secondary causes of hepatic fat accumulation (1). It is estimated that the global prevalence of NAFLD was approximately 30% in 2019, and this figure continues to rise, the prevalence of NAFLD is relatively high among middle-aged and elderly populations (2). Additionally, Nonalcoholic steatohepatitis (NASH) represents a more severe stage of liver injury in NAFLD. NASH is often accompanied by metabolic syndromes such as obesity and type 2 diabetes, and the interplay among them makes the disease burden related to NASH even more severe (3). A study conducted in Texas, USA, reported that 38% of asymptomatic middle-aged individuals were diagnosed with NAFLD, while 14% were diagnosed with NASH and 35% of individuals with NASH exhibited signs of liver fibrosis (4). Pathologically, NASH is typically characterized by HS, lobular inflammation, and ballooning degeneration, with or without perisinusoidal fibrosis (5). Additionally, NASH may progress to more severe conditions, including liver fibrosis, liver cirrhosis, liver failure, and liver cancer (6, 7). The disease burden caused by NASH deserves more attention. Notably, approximately 20% of patients with NASH progress to cirrhosis and were at high risk of developing hepatocellular carcinoma (HCC), with multiple factors driving this progression (8).

Among these factors, high fasting blood glucose and metabolic risks stood out as the most critical. They played a vital role in promoting the progression of NASH to NASH-related liver cancer (NALC) through diverse mechanisms, including chronic inflammation, oxidative stress, metabolic disturbances, and imbalances between cellular apoptosis and proliferation. Specifically, hyperglycemia induced endothelial dysfunction, triggers increased secretion of vascular endothelial growth factor (VEGF), upregulates inflammatory genes, and exacerbates oxidative stress—all of which contributed to hepatocyte proliferation and NALC development (8). Additionally, in a hyperglycemic environment, adiponectin deficiency impaired its tumor-suppressive apoptotic function mediated by the JNK/caspase-3 pathway, accelerated liver fibrosis, and synergistically facilitated NALC progression. Moreover, metabolic risks such as obesity-induced inflammation and high-fat diets further amplified these pathological processes: they accelerated intestinal microbiota dysbiosis, increased intestinal mucosal permeability, and caused leakage of inflammatory factors, thereby inducing liver inflammation, tissue damage, and DNA injury—ultimately driving NASH progression and the development of metabolic disease-related HCC (9). A large retrospective cohort study demonstrated that compared with obese patients with NAFLD, the risk of HCC development was 2.6 times higher when obesity, diabetes, hypertension, and dyslipidemia (key components of metabolic risk) coexisted (10).

Nowadays we continue to face significant health challenges from liver cancer, which is the sixth most common cancer globally and ranks third in cancer-related mortality. It is estimated that by 2025, over 1 million individuals will be diagnosed with liver cancer each year (11). Among U.S. liver transplant candidates with HCC, NAFLD related HCC has emerged as a leading cause (12). Additionally, NASH is increasingly recognized as the fastest-growing contributor to liver cancer mortality. From 1990 to 2019, the global mortality rate of NAFLD related liver cancer among adults aged over the age of 55 increased by 7.86%, imposing the greatest disease burden (13). Aging was a pivotal driver in the pathogenesis of NAFLD and NASH, significantly elevating the risk of liver disease in the elderly through multifaceted mechanisms. Aging disrupted hepatic homeostasis by impairing lipid metabolism, mitochondrial function, and autophagy, concurrent with systemic alterations including insulin/leptin resistance, reduced growth hormone/adiponectin, hypercholesterolemia, DNA damage, ROS accumulation, and telomere shortening. These cumulative changes rendered the elderly more susceptible to developing NASH and advanced fibrosis (14). Furthermore, chronic moderate liver injury in this population promoted compensatory hepatocyte proliferation, increasing the risk of HCC (15). Preclinical models indicated that aging accelerated HCC progression by altering the tumor microenvironment via the senescence-associated secretory phenotype (SASP) (16). Clinical evidence corroborated this heightened risk: the prevalence of NAFLD was approximately 16% higher in elderly patients, who also demonstrated a greater propensity for advanced fibrosis and severe liver damage in NASH (17). Critically, a large cohort study revealed a pronounced age-related increase in NAFLD-associated HCC incidence (0.01, 0.21, and 0.41 per 1, 000 person-years for ages < 45, 45–64, and >65 years, respectively) (18). Consequently, focused research on this high-risk population is imperative. Previous studies have primarily focused on analyzing the global disease burden or the burden of NALC in specific countries, without paying particular attention to the disease burden in middle-aged and the older population, which is a key demographic group (13, 19, 20).

Therefore, in this study we used the global burden of disease (GBD) database to systematically and comprehensively analyze trends in the incidence, prevalence, mortality, and disability-adjusted life years (DALYs) of NALC among adults over 45 years old from 1990 to 2021. The disease burden and epidemiological trends of NALC were assessed at global, regional, and national levels. Additionally, frontier analysis was used to estimate the best achievable DALYs for NALC based on the level of Socio-demographic Index (SDI) and projected the disease burden trends through 2050. This research will contribute to a deeper understanding of the epidemiological characteristics of NALC in key populations, facilitate the more effective allocation of healthcare resources, and support policymakers in developing targeted strategies to address the burden of NALC, particularly in high-risk regions.

## 2 Methods

### 2.1 Data source

The research data were obtained from the GBD 2021 database, published by the Institute for Health Metrics and Evaluation (IHME) at the University of Washington, USA.^1^ This database employed modeling based on publicly available data to estimate the burden of 371 diseases and injuries across 204 countries and territories from 1990 to 2021, stratified by age and sex. Additionally, it includes data from five different SDI country groups and 21 GBD regions. The SDI, introduced by IHME in 2015, is a composite measure used to assess the development level of a country or region and to reflect the relationship between social development and population health outcomes. It is calculated as the geometric mean of three indicators: the average years of education among individuals aged 15 years and older, the total fertility rate among individuals under 25 years old, and the lag-distributed income per capita. The SDI ranges from 0 to 1, where 0 represents the lowest income, the lowest level of education, and the highest fertility rate, while 1 indicates the opposite (13). The 21 GBD regions are classified based on countries and territories with similar characteristics, such as epidemiological profiles, causes of mortality, and geographical proximity. NALC referred to primary liver cancer arising from NASH progression. While the vast majority of NASH-driven malignancies were hepatocellular carcinomas, cholangiocarcinoma or combined hepatocellular-cholangiocarcinoma occur rarely (21). In the United States, NASH has emerged as the second leading cause of HCC-related liver transplantation (22). High fasting plasma glucose was defined as serum fasting plasma glucose greater than 5.4 mmol/L. We quantified metabolic risks according to GBD criteria, including high LDL cholesterol, high systolic blood pressure, high BMI, low bone mineral density, and impaired kidney function (23).

### 2.2 Joinpoint regression analysis

The basic concept of the Joinpoint regression analysis model is to divide a long-term trend line into several segments by time points, with each segment described by a fitted continuous straight line. In this study, we applied the Joinpoint regression model to log-transform age-standardized incidence rates, prevalence rates, mortality rates, and DALYs of diseases. Standard errors were approximated based on the binomial distribution, and we analyzed the Annual Percent Change (APC), Average Annual Percent Change (AAPC), and their 95% confidence intervals (CIs). In our study, APC means the trend of the disease burden of NALC during a specific period, while AAPC represents the overall trend of the disease burden over a longer time frame. An AAPC or APC less than 0 indicates a decreasing trend, greater than 0 indicates an increasing trend, and inclusion of 0 suggests a stable trend. In our study, Joinpoint software (Version 5.4.0) was employed to conduct the Joinpoint regression analysis.

### 2.3 Frontier analysis

Frontier analysis evaluates and optimizes the performance of health systems using Data Envelopment Analysis (DEA) and Local Polynomial Regression (LOESS). When examining the relationship between disease burden and socio-demographic development, frontier analysis demonstrates significant advantages and practicality. It helps identify the minimum possible burden, measures efficiency differences, and assesses the unrealized health potential of countries at their respective development levels. This aids in optimizing health resource allocation to support policy formulation and improvement. The effective difference between a country and the frontier is defined by the gap between its observed and potentially achievable disease burden. This gap can be reduced or eliminated by leveraging the country’s socio-demographic resources.

### 2.4 Bayesian age-period-cohort models

The Bayesian Age-Period-Cohort (BAPC) model addresses the collinearity issues inherent in traditional age-period-cohort models by introducing prior distributions and regularization techniques. Using the Integrated Nested Laplace Approximation (INLA), the BAPC model avoids the mixing and convergence problems associated with Markov Chain Monte Carlo (MCMC) sampling methods. As a result, the BAPC model is often employed for predictive analysis.

### 2.5 Statistical analysis

We selected adults aged 45 years and older diagnosed with NALC as the study population, stratified into five-year age groups (45–49, 50–54, 55–59, 60–64, 65–69, 70–74, 75–79, 80–84, 85–90, and 90+ years) and further analyzed by sex (male and female). Estimates of incidence, prevalence, mortality, and DALYs, along with their 95% uncertainty intervals (UI), were utilized. Age-standardized incidence, prevalence, mortality, and DALYs were calculated based on the age structure of the GBD world standard population. The Joinpoint regression model was employed to analyze temporal trends from 1990 to 2021, estimating AAPC and APC for specific time periods. Frontier analysis was conducted to determine the minimum achievable age-standardized DALYs based on varying levels of development. The BAPC model was used to project the disease burden of NALC among adults aged 45 and older from 2022 to 2050. Data analysis for the global burden of NALC among adults aged 45 and older from 1990 to 2021 was performed using Excel 2021 and R software. Statistical analysis and visualization were conducted using R packages such as dplyr, officer, BAPC, and ggplot2. *p* < 0.05 was considered statistically significant.

## 3 Results

### 3.1 Global burdens of NALC in adults aged 45 and above from 1990 to 2021

The Age-standardized incidence rate of NALC among adults aged over 45 in 2021 was 1.69 (95% UI: 1.18, 2.32) per 100, 000 population, the Age-standardized prevalence rate was 2.01 (95% UI: 1.41, 2.77) per 100, 000 population, the Age-standardized mortality rate was 1.66 (95% UI: 1.16, 2.28) per 100, 000 population, and the Age-standardized DALYs was 36.69 (95% UI: 25.62, 50.61) per 100, 000 person-years. This indicates that in 2021, the number of newly diagnosed middle-aged and elderly patients was 39720.95 (95% UI: 27741.64, 54516.22), with 20412.94 (95% UI: 13980.65, 28639.64) in men and 19308.00 (95% UI: 13465.02, 26263.86) in women. The total number of prevalent cases in 2021 was 47791.46 (95% UI: 33510.90, 65696.92), with 25644.38 (95% UI: 17579.47, 35901.25) in men and 22147.08 (95% UI: 15430.42, 30352.20) in women. The number of deaths from the disease among middle-aged and elderly individuals in 2021 was 38795.79 (95% UI: 27082.97, 53287.18), with 19375.91 (95% UI: 13218.43, 27183.80) in men and 19, 419.88 (95% UI: 13, 539.34, 26461.13) in women. The DALYs resulting from this disease was 877353.23 (95% UI: 612675.19, 1209993.51) person-years, with 461253.83 (95% UI: 313352.93, 650005.76) person-years in men and 416099.41 (95% UI: 291670.12, 565933.55) person-years in women (Supplementary Table 1). From 1990 to 2021, the Estimated Annual Percentage Change (EAPC) for all disease burden indicators in adults aged over 45 was positive, indicating an overall increasing trend in disease burden among middle-aged and elderly populations globally (Table 1). Globally, the age-standardized DALYs are highest among individuals aged 80–84 years, the age-standardized prevalence and incidence rates are highest among those aged 85–89 years, and the age-standardized mortality rate is highest among individuals aged 90–94 years (Supplementary Table 2).

**Table 1:** Age-standardized NALC burden results among adults aged 45 and older for the global, five SDI regions, and 21 GBD regions.

Location	Incidence			Prevalence			Deaths			DALYs		
	1990 (per 100, 000 population, 95%UI)	2021 (per 100, 000 population, 95%UI)	EAPCs (95%CI)	1990 (per 100, 000 population, 95%UI)	2021 (per 100, 000 population, 95%UI)	EAPCs (95%CI)	1990 (per 100, 000 population, 95%UI)	2021 (per 100, 000 population, 95%UI)	EAPCs (95%CI)	1990 (per 100, 000 population, 95%UI)	2021 (per 100, 000 population, 95%UI)	EAPCs (95%CI)
Global	1.22 (0.84, 1.70)	1.69 (1.18, 2.32)	1.05 (0.97, 1.14)	1.29 (0.89, 1.78)	2.01 (1.41, 2.77)	1.50 (1.38, 1.63)	1.28 (0.88, 1.79)	1.66 (1.16, 2.28)	0.86 (0.77, 0.94)	30.15 (20.63, 42.19)	36.69 (25.62, 50.61)	0.63 (0.55, 0.72)
SDI												
High SDI	1.11 (0.77, 1.55)	1.83 (1.25, 2.56)	1.43 (1.19, 1.67)	1.39 (0.97, 1.90)	2.81 (1.91, 3.92)	2.21 (1.82, 2.60)	1.06 (0.73, 1.50)	1.55 (1.05, 2.18)	1.13 (0.92, 1.33)	24.29 (16.61, 34.36)	32.35 (22.08, 45.37)	0.82 (0.63, 1.01)
High-middle SDI	1.00 (0.69, 1.36)	1.33 (0.92, 1.84)	1.06 (0.92, 1.19)	1.02 (0.71, 1.39)	1.54 (1.06, 2.14)	1.52 (1.39, 1.66)	1.06 (0.74, 1.46)	1.31 (0.90, 1.81)	0.78 (0.66, 0.90)	25.42 (17.66, 34.82)	29.18 (20.23, 40.60)	0.51 (0.38, 0.64)
Middle SDI	1.43 (1.00, 1.98)	1.77 (1.24, 2.43)	0.88 (0.76, 1.01)	1.41 (0.98, 1.94)	1.92 (1.34, 2.65)	1.22 (1.07, 1.37)	1.56 (1.09, 2.15)	1.81 (1.28, 2.48)	0.66 (0.54, 0.77)	36.35 (25.33, 50.12)	40.04 (28.12, 55.04)	0.45 (0.34, 0.57)
Low-middle SDI	1.13 (0.72, 1.74)	1.58 (1.08, 2.21)	1.06 (0.96, 1.16)	1.10 (0.70, 1.68)	1.55 (1.06, 2.17)	1.12 (1.02, 1.22)	1.24 (0.79, 1.91)	1.72 (1.18, 2.40)	1.01 (0.89, 1.12)	29.17 (18.65, 44.57)	39.87 (27.20, 55.78)	0.95 (0.86, 1.05)
Low SDI	2.18 (1.20, 3.67)	2.11 (1.28, 3.24)	−0.31 (−0.39, −0.24)	2.04 (1.13, 3.42)	1.99 (1.20, 3.06)	−0.29 (−0.37, −0.22)	2.42 (1.33, 4.10)	2.36 (1.43, 3.61)	−0.29 (−0.37, −0.21)	54.61 (30.36, 91.50)	51.80 (31.11, 80.01)	−0.39 (−0.48, −0.30)
GBD 21 regions												
Andean Latin America	0.90 (0.49, 1.48)	1.17 (0.64, 1.91)	0.62 (0.34, 0.90)	0.84 (0.46, 1.37)	1.09 (0.59, 1.80)	0.68 (0.41, 0.96)	1.02 (0.56, 1.67)	1.31 (0.72, 2.15)	0.56 (0.25, 0.86)	21.13 (11.51, 34.67)	26.05 (14.02, 42.88)	0.44 (0.15, 0.74)
Australasia	0.52 (0.31, 0.81)	2.11 (1.24, 3.32)	4.56 (4.33, 4.79)	0.58 (0.35, 0.90)	2.99 (1.76, 4.70)	5.69 (5.32, 6.07)	0.53 (0.31, 0.83)	1.91 (1.11, 3.02)	4.12 (3.94, 4.30)	11.91 (7.03, 18.68)	41.50 (24.25, 65.51)	4.06 (3.91, 4.21)
Caribbean	0.66 (0.37, 1.07)	0.72 (0.41, 1.17)	−0.10 (−0.37, 0.18)	0.63 (0.36, 1.02)	0.72 (0.41, 1.18)	0.08 (−0.18, 0.35)	0.74 (0.42, 1.19)	0.79 (0.44, 1.27)	−0.12 (−0.42, 0.18)	15.53 (8.75, 25.02)	16.86 (9.44, 27.39)	−0.05 (−0.34, 0.24)
Central Asia	1.70 (0.95, 2.79)	1.95 (1.09, 3.20)	0.41 (0.32, 0.51)	1.67 (0.93, 2.73)	1.88 (1.05, 3.08)	0.34 (0.25, 0.43)	1.85 (1.03, 3.04)	2.14 (1.20, 3.53)	0.39 (0.29, 0.50)	43.98 (24.54, 71.91)	47.92 (26.57, 78.64)	0.13 (0.04, 0.22)
Central Europe	0.87 (0.51, 1.39)	0.95 (0.58, 1.44)	0.32 (0.16, 0.49)	0.82 (0.48, 1.32)	0.96 (0.58, 1.45)	0.56 (0.41, 0.72)	0.97 (0.57, 1.55)	1.04 (0.64, 1.57)	0.27 (0.06, 0.47)	20.66 (12.16, 33.26)	21.87 (13.40, 33.15)	0.25 (0.05, 0.45)
Central Latin America	0.82 (0.53, 1.22)	1.04 (0.69, 1.48)	0.96 (0.69, 1.23)	0.78 (0.50, 1.16)	1.00 (0.66, 1.43)	1.03 (0.76, 1.30)	0.91 (0.59, 1.36)	1.15 (0.76, 1.64)	0.87 (0.57, 1.17)	19.73 (12.64, 29.34)	24.07 (15.92, 34.19)	0.77 (0.47, 1.08)
Central Sub-Saharan Africa	1.67 (0.57, 4.16)	1.41 (0.47, 3.70)	−0.95 (−1.11, −0.78)	1.58 (0.54, 3.90)	1.33 (0.45, 3.47)	−0.93 (−1.09, −0.76)	1.85 (0.61, 4.66)	1.57 (0.52, 4.21)	−0.90 (−1.06, −0.74)	43.28 (14.84, 107.23)	35.31 (11.98, 92.77)	−1.03 (−1.19, −0.87)
East Asia	1.56 (1.10, 2.13)	1.85 (1.26, 2.58)	0.94 (0.76, 1.13)	1.57 (1.10, 2.14)	2.19 (1.49, 3.08)	1.50 (1.32, 1.69)	1.68 (1.18, 2.28)	1.76 (1.21, 2.45)	0.55 (0.36, 0.74)	40.45 (28.27, 55.09)	38.91 (26.61, 54.66)	0.17 (−0.01, 0.35)
Eastern Europe	0.44 (0.32, 0.59)	0.72 (0.53, 0.96)	1.69 (1.43, 1.95)	0.44 (0.32, 0.58)	0.72 (0.52, 0.96)	1.74 (1.47, 2.01)	0.48 (0.35, 0.64)	0.79 (0.57, 1.04)	1.76 (1.46, 2.06)	11.21 (8.15, 14.84)	17.38 (12.62, 23.12)	1.56 (1.26, 1.86)
Eastern Sub-Saharan Africa	2.39 (1.39, 3.89)	2.51 (1.47, 3.98)	−0.14 (−0.26, −0.03)	2.23 (1.30, 3.62)	2.35 (1.37, 3.72)	−0.11 (−0.22, −0.01)	2.66 (1.54, 4.34)	2.81 (1.64, 4.42)	−0.11 (−0.22, 0.00)	59.85 (34.97, 97.41)	61.45 (35.71, 97.35)	−0.21 (−0.33, −0.09)
High-income Asia Pacific	2.76 (1.92, 3.88)	2.29 (1.46, 3.43)	−1.21 (−1.57, −0.84)	3.79 (2.70, 5.16)	4.31 (2.73, 6.42)	−0.06 (−0.53, 0.41)	2.52 (1.73, 3.59)	1.72 (1.11, 2.54)	−1.71 (−2.09, −1.32)	59.63 (40.89, 85.60)	34.34 (21.85, 51.63)	−2.30 (−2.67, −1.92)
High-income North America	0.78 (0.58, 1.00)	2.07 (1.52, 2.70)	3.26 (3.05, 3.47)	0.90 (0.67, 1.16)	2.89 (2.12, 3.77)	3.99 (3.64, 4.33)	0.72 (0.54, 0.93)	1.74 (1.26, 2.27)	2.97 (2.81, 3.13)	15.22 (11.39, 19.59)	37.03 (27.18, 48.18)	3.09 (2.90, 3.28)
North Africa and Middle East	1.37 (0.69, 2.56)	2.22 (1.32, 3.46)	1.55 (1.31, 1.78)	1.31 (0.67, 2.44)	2.22 (1.31, 3.47)	1.68 (1.46, 1.90)	1.51 (0.76, 2.85)	2.41 (1.42, 3.73)	1.43 (1.18, 1.69)	34.26 (17.40, 63.66)	54.12 (31.49, 84.33)	1.42 (1.21, 1.63)
Oceania	1.25 (0.53, 2.82)	1.14 (0.55, 2.25)	−0.51 (−0.69, −0.33)	1.21 (0.51, 2.72)	1.13 (0.53, 2.23)	−0.43 (−0.61, −0.24)	1.37 (0.59, 3.11)	1.24 (0.59, 2.44)	−0.54 (−0.71, −0.36)	32.07 (13.62, 72.98)	28.67 (13.47, 57.70)	−0.55 (−0.73, −0.37)
South Asia	0.77 (0.56, 1.02)	1.20 (0.89, 1.57)	1.54 (1.44, 1.64)	0.75 (0.54, 0.99)	1.17 (0.86, 1.53)	1.52 (1.43, 1.60)	0.83 (0.60, 1.10)	1.32 (0.98, 1.71)	1.52 (1.40, 1.64)	20.07 (14.52, 26.65)	29.94 (22.04, 39.12)	1.29 (1.19, 1.38)
Southeast Asia	1.91 (1.15, 2.98)	2.23 (1.31, 3.51)	0.47 (0.35, 0.58)	1.88 (1.13, 2.93)	2.31 (1.36, 3.63)	0.64 (0.52, 0.75)	2.09 (1.26, 3.26)	2.38 (1.41, 3.73)	0.40 (0.29, 0.51)	48.82 (29.53, 76.16)	53.37 (31.58, 83.43)	0.24 (0.13, 0.35)
Southern Latin America	0.23 (0.13, 0.37)	0.64 (0.36, 1.03)	3.99 (3.79, 4.19)	0.22 (0.12, 0.36)	0.66 (0.37, 1.06)	4.19 (4.00, 4.39)	0.25 (0.14, 0.41)	0.69 (0.38, 1.10)	3.93 (3.68, 4.17)	5.45 (3.01, 8.96)	14.74 (8.21, 23.58)	3.85 (3.60, 4.09)
Southern Sub-Saharan Africa	1.96 (1.05, 3.23)	3.47 (2.39, 4.81)	1.38 (0.80, 1.96)	1.86 (0.99, 3.07)	3.23 (2.22, 4.48)	1.35 (0.80, 1.89)	2.18 (1.18, 3.61)	3.88 (2.69, 5.38)	1.35 (0.75, 1.96)	48.14 (25.79, 79.88)	84.28 (57.67, 117.45)	1.36 (0.74, 1.98)
Tropical Latin America	0.41 (0.30, 0.53)	0.53 (0.39, 0.69)	1.52 (1.21, 1.84)	0.39 (0.29, 0.50)	0.52 (0.38, 0.68)	1.65 (1.34, 1.95)	0.45 (0.34, 0.59)	0.58 (0.43, 0.76)	1.55 (1.23, 1.87)	9.96 (7.44, 12.91)	12.77 (9.41, 16.58)	1.58 (1.28, 1.88)
Western Europe	0.70 (0.44, 1.05)	1.24 (0.76, 1.89)	1.99 (1.85, 2.14)	0.77 (0.49, 1.15)	1.83 (1.13, 2.78)	3.12 (2.83, 3.41)	0.72 (0.46, 1.09)	1.13 (0.70, 1.73)	1.55 (1.43, 1.66)	15.26 (9.64, 22.94)	23.34 (14.36, 35.51)	1.48 (1.35, 1.60)
Western Sub-Saharan Africa	3.96 (1.99, 7.19)	3.81 (2.38, 5.74)	−0.40 (−0.49, −0.31)	3.72 (1.86, 6.73)	3.54 (2.20, 5.37)	−0.42 (−0.51, −0.33)	4.40 (2.22, 8.00)	4.27 (2.68, 6.43)	−0.36 (−0.45, −0.27)	98.50 (49.51, 178.38)	91.41 (56.72, 139.19)	−0.50 (−0.60, −0.40)

### 3.2 Regional burdens of NALC in adults aged 45 and above from 1990 to 2021

In the five SDI regions, the Low SDI region exhibited the highest Age-standardized incidence rate [2.11 (95% UI: 1.28, 3.24)], Age-standardized mortality rate [2.36 (95% UI: 1.43, 3.61)], and Age-standardized DALYs [51.80 (95% UI: 31.11, 80.01)] for NALC in 2021, whereas the High SDI region had the highest Age-standardized prevalence rate [2.81 (95% UI: 1.91, 3.92)]. In terms of temporal trends, NALC disease burden increased in all SDI regions except for the Low SDI region. Notably, the High SDI region showed the most pronounced increases in Age-standardized incidence rate (EAPC = 1.43, 95% CI: 1.19, 1.67), Age-standardized prevalence rate (EAPC = 2.21, 95% CI: 1.82, 2.60), and Age-standardized mortality rate (EAPC = 1.13, 95% CI: 0.92, 1.33), while the Low-Middle SDI region showed the most significant increase in Age-standardized DALYs (EAPC = 0.95, 95% CI: 0.86, 1.05) (Table 1).

Among the 21 GBD regions, Western Sub-Saharan Africa had the highest Age-standardized incidence rate [3.81 (95% UI: 2.38, 5.74)], Age-standardized mortality rate [4.27 (95% UI: 2.68, 6.43)], and Age-standardized DALYs [91.41 (95% UI: 56.72, 139.19)], while High-income Asia Pacific had the highest Age-standardized prevalence rate [4.31 (95% UI: 2.73, 6.42)], and Tropical Latin America had the lowest disease burden. In terms of temporal trends, Australasia, Southern Latin America, and High-income North America experienced the most significant increases in disease burden (Table 1).

### 3.3 National or regional burdens of NALC in adults aged 45 and above from 1990 to 2021

**Figure 1:**
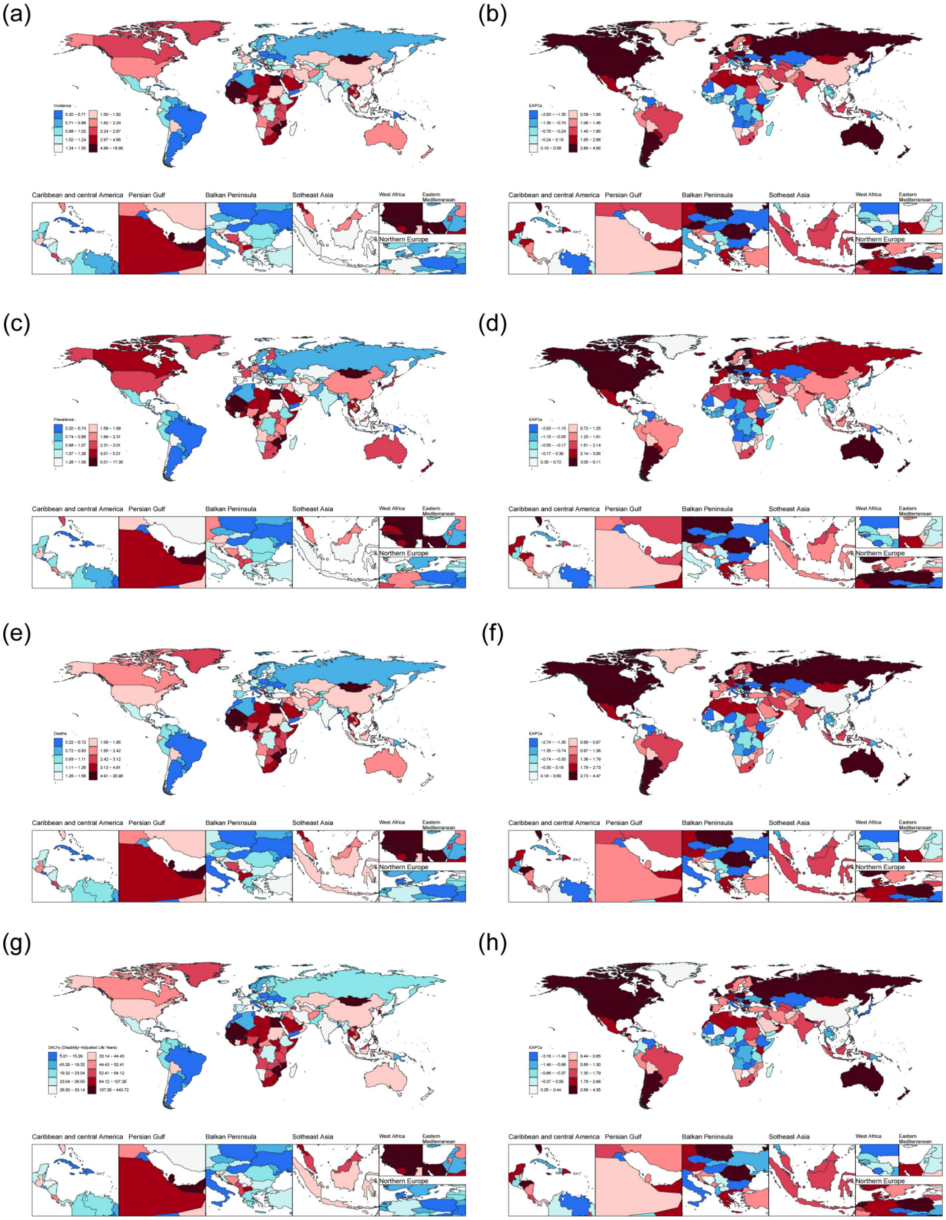
The disease burden of NALC among adults aged 45 years and older across 204 countries and regions in 2021. **(a)** Age-standardized incidence rate. **(b)** EAPC of Age-standardized incidence rate. **(c)** Age-standardized prevalence rate. **(d)** EAPC of Age-standardized prevalence rate. **(e)** Age-standardized mortality rate. **(f)** EAPC of Age-standardized mortality rate. **(g)** Age-standardized DALYs. **(h)** EAPC of Age-standardized DALYs.

In 2021, among adults aged 45 years and older, the highest Age-standardized incidence rate of NALC was in Islamic Republic of Mauritania [9.74 (95%UI: 4.26, 18.50)], the highest Age-standardized prevalence rate was in Republic of Mozambique [9.40 (95%UI: 3.67, 20.06)], the highest Age-standardized mortality rate was in State of Qatar [9.67 (95%UI: 4.84, 17.53)], the highest Age-standardized DALYs was in Federal Republic of Somalia. Conversely, the lowest Age-standardized incidence rate [0.20 (95%UI: 0.09, 0.37)], Age-standardized prevalence rate [0.20 (95%UI: 0.09, 0.37)], Age-standardized mortality rate [0.22 (95%UI: 0.10, 0.40)] was reported in Kingdom of Morocco, while the lowest Age-standardized DALYs was in Republic of Haiti [10.28 (95%UI: 3.69, 24.06)]. From 1990 to 2021, the five countries or regions with the largest increase in Age-standardized incidence rate of NALC among adults aged 45 years and older were the United Kingdom of Great Britain and Northern Ireland, Australia, the Eastern Republic of Uruguay, the Republic of Poland, and Taiwan (Province of China). The greatest increases in Age-standardized prevalence rate were observed in Australia, the United Kingdom of Great Britain and Northern Ireland, Taiwan (Province of China), the Eastern Republic of Uruguay, and New Zealand. The highest increases in Age-standardized mortality rate were recorded in the United Kingdom of Great Britain and Northern Ireland, Australia, the Eastern Republic of Uruguay, the Republic of Poland, and Romania. Similarly, the five countries or regions with the largest increase in Age-standardized DALYs were Australia, the United Kingdom of Great Britain and Northern Ireland, the Republic of Poland, the Eastern Republic of Uruguay, and Romania (Figure 1; Supplementary Table 3).

### 3.4 Global trends of NALC from 1990 to 2021

**Figure 2:**
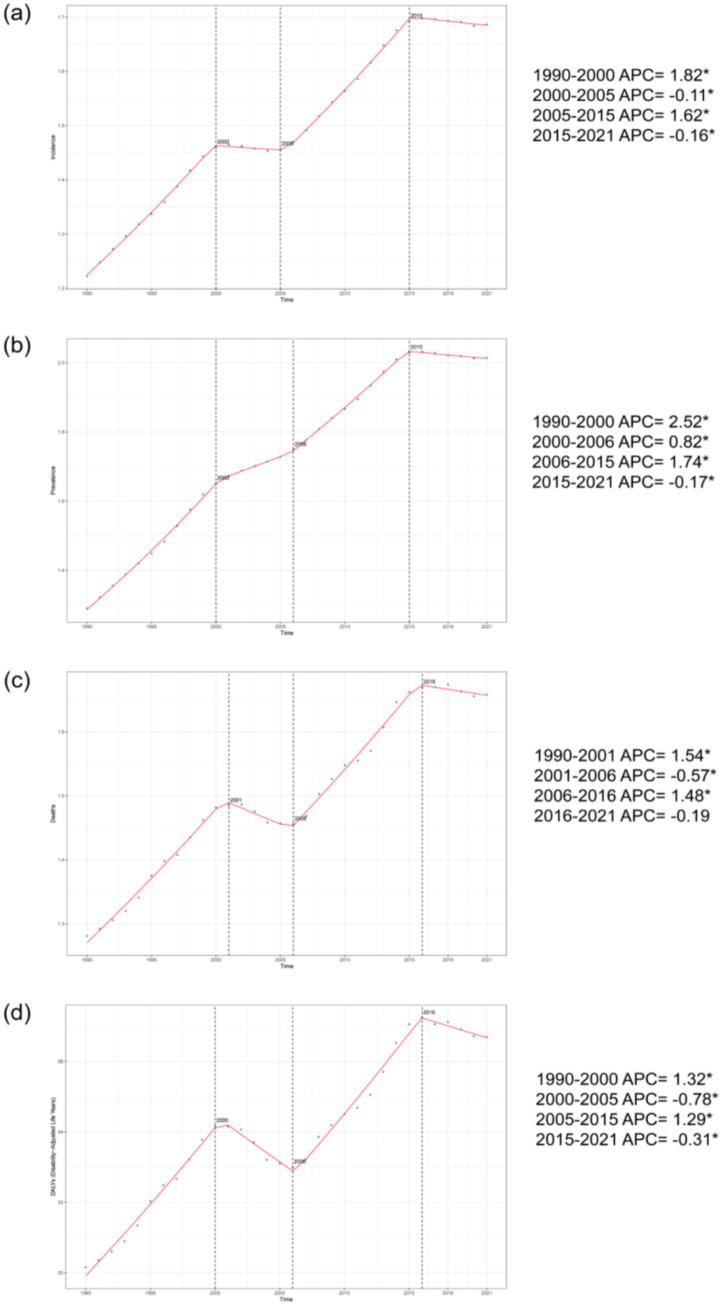
The percentage change trends in the incidence rate, prevalence rate, mortality rate, and DALYs of NALC among adults aged 45 and older globally from 1990 to 2021. **(a)** represents the incidence rate, **(b)** represents the prevalence rate, **(c)** represents the mortality rate, and **(d)** represents DALYs. ^*^p < 0.05.

Joinpoint regression analysis reveals that from 1990 to 2021, the age-standardized incidence rate, prevalence rate, mortality rate, and DALYs for adults aged 45 and older showed significant upward trends globally. Specifically, AAPC for the age-standardized incidence rate was 0.015 (0.015, 0.015), for the age-standardized prevalence rate was 0.024 (0.023, 0.024), for the age-standardized mortality rate was 0.013 (0.012, 0.013), and for age-standardized DALYs was 0.219 (0.208, 0.230). The years of significant change for these four disease burden indicators were concentrated around 2000 (with mortality in 2001), 2006 (with incidence in 2005), and 2015, as shown in Figure 2. Since 2016, all disease burden indicators, except for mortality, have exhibited slight declines.

### 3.5 Association between disease burden and SDI

**Figure 3:**
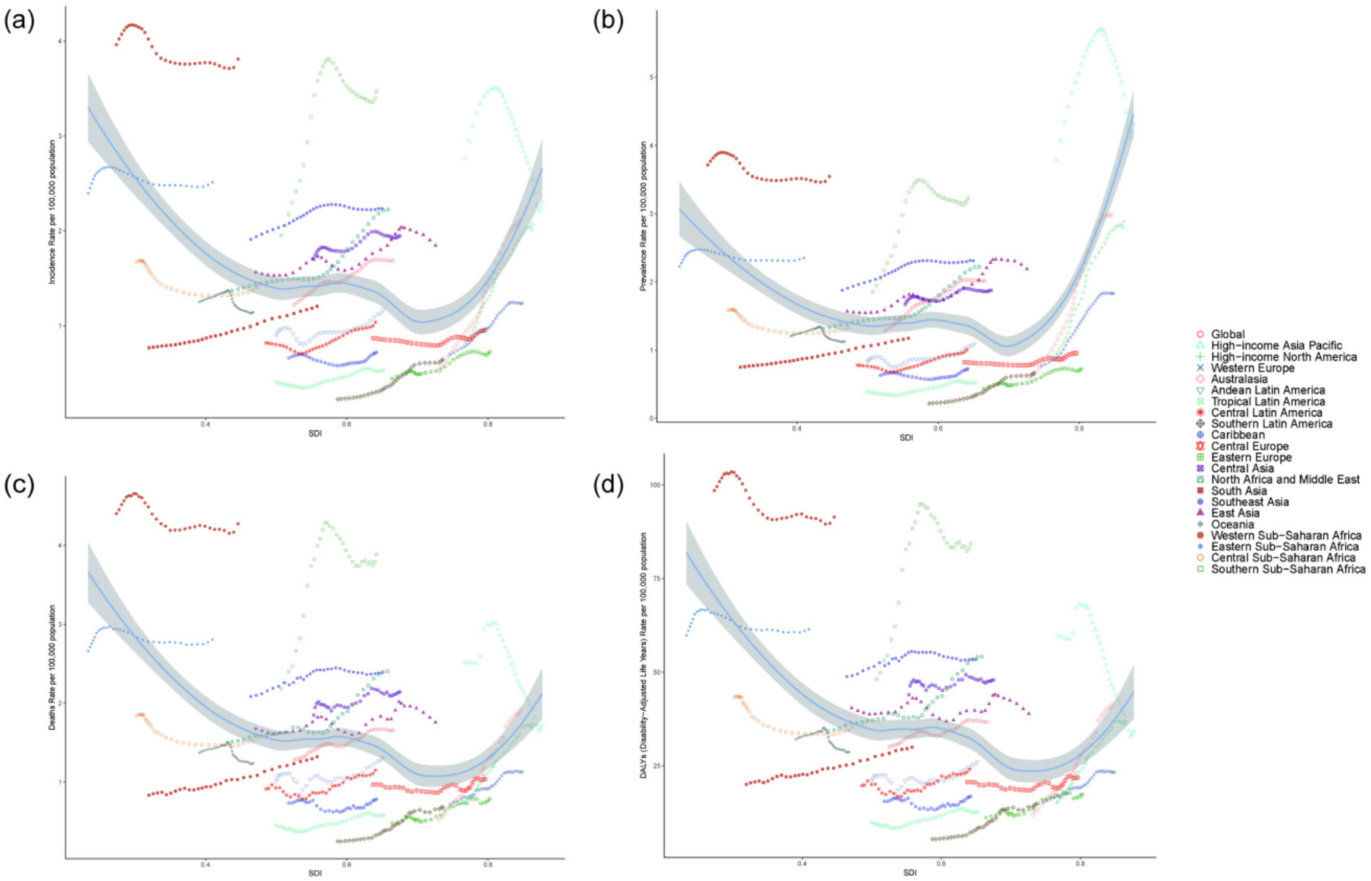
The association between the disease burden of NALC among adults aged 45 and older and the SDI. **(a)** Incidence rate across 21 regions, **(b)** Prevalence rate across 21 regions, **(c)** Mortality rate across 21 regions, **(d)** DALYs across 21 regions.

In the 21 GBD regions, the SDI exhibits a nonlinear relationship with the Age-standardized incidence rate, prevalence rate, mortality rate, and DALYs of NALC among adults aged 45 and older. Specifically, as SDI increases, the disease burden of NALC initially declines, reaching its lowest point when SDI is approximately 0.70, after which it begins to rise. This pattern suggests that both low-SDI and high-SDI regions experience a higher disease burden. Notably, Australasia has the fastest-growing disease burden among the 21 GBD regions (Figure 3).

### 3.6 Frontier analysis

**Figure 4:**
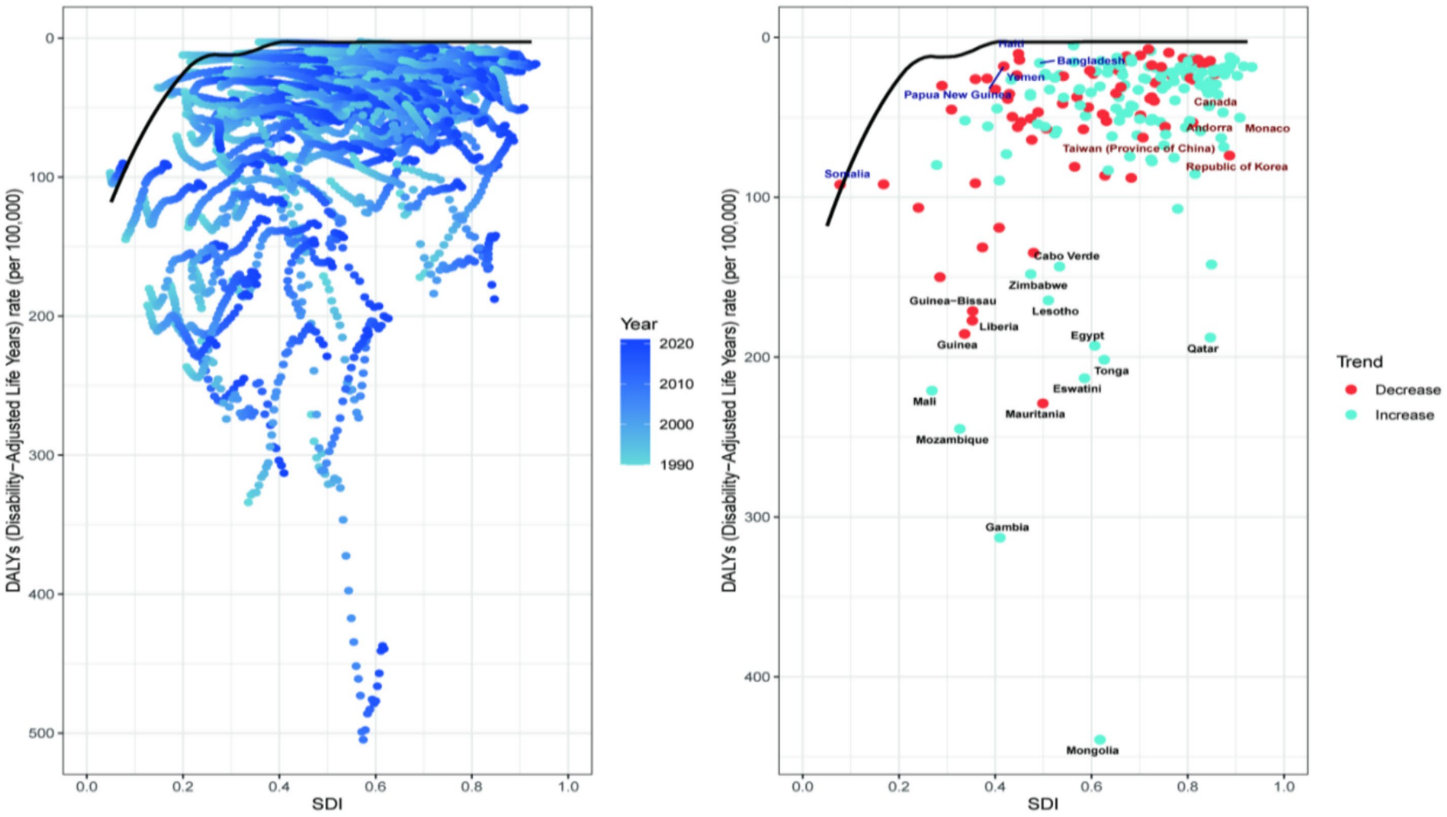
Frontier analysis of 204 countries and 21 regions. Each point represents a country, while the line represents the frontier, indicating the lowest disease burden. The color gradient from light blue to dark blue reflects changes in the NALC disease burden from 1990 to 2021. Points with black text represent the 15 countries or regions with the greatest deviation from the frontier. Points with blue text denote the five low-SDI countries (SDI < 0.50) with the smallest deviation from the frontier. Points with red text also indicate the five high-SDI countries (SDI > 0.85) with the greatest deviation from the frontier.

To analysis the trends in the disease burden of NALC among adults aged 45 and above across different regions and to explore potential areas for improvement, a frontier analysis was conducted based on Age-standardized DALYs across 204 countries or regions from 1990 to 2021 (shown in Figure 4). The 15 countries or regions with the greatest potential for improvement are Mongolia (effective difference: 436.64), Gambia (effective difference: 310.00), Mozambique (effective difference: 233.69), Mauritania, Eswatini (effective difference: 210.48), Mali (effective difference: 208.78), Tonga (effective difference: 198.97), Egypt (effective difference: 190.20), Qatar (effective difference: 185.11), Guinea (effective difference: 174.47), Liberia (effective difference: 166.49), Lesotho (effective difference: 161.59), Guinea-Bissau (effective difference: 160.50), Zimbabwe (effective difference: 145.17). These countries exhibit a disproportionately high disease burden relative to their corresponding SDI. Conversely, countries with low SDI and low effective differences include Somalia, Papua New Guinea, Yemen, Malta, and Bangladesh. High-performing countries or regions with high SDI include Canada, Andorra, Monaco, Taiwan (Province of China), and the Republic of Korea. Other disease burden indicators, such as age-standardized incidence, prevalence, mortality, and DALYs, are consistent, as detailed in Supplementary Figure 1.

### 3.7 Projected disease burden through 2050

**Figure 5:**
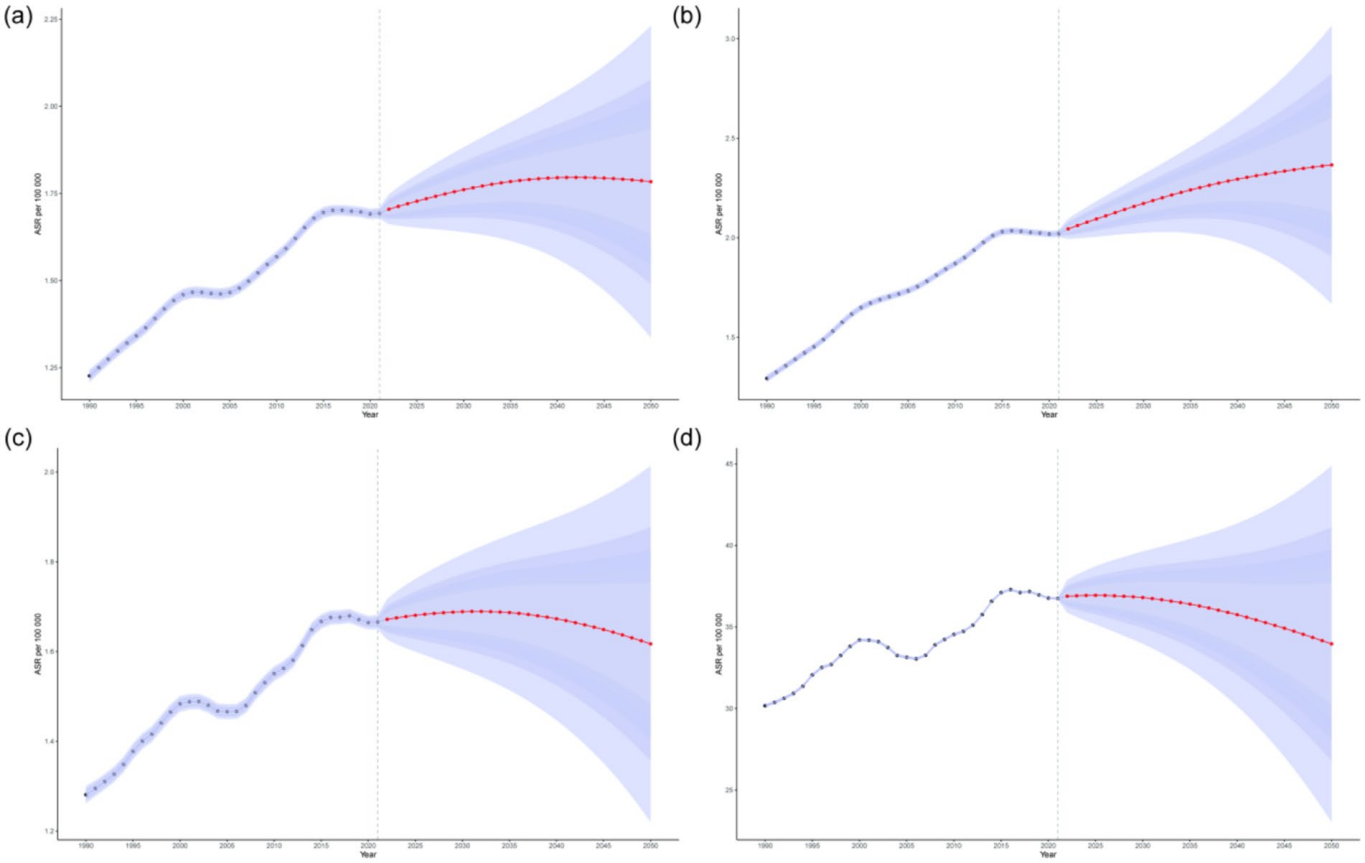
Projections of the age-standardized incidence rate **(a)**, prevalence rate **(b)**, mortality rate **(c)**, and DALYs **(d)** of NALC among adults aged 45 and older by 2050. The red line represents the predicted values, while the blue shaded area indicates the 95% UI range.

By using BAPC model, future projections of the age-standardized incidence rate, prevalence rate, mortality rate, and DALYs of NALC have been estimated. Globally, among adults aged 45 and older, the age-standardized incidence rate, mortality rate, and DALYs are projected to initially increase and then decline between 2022 and 2050, whereas the age-standardized prevalence rate is expected to show a continuous upward trend, reaching 2.37 (95% UI: 1.67, 3.06) by 2050. Specifically, the age-standardized incidence rate is projected to peak at 1.80 (95% UI: 1.53, 2.06) in 2042, while the age-standardized prevalence rate is expected to reach 2.35 (95% UI: 1.79, 2.90) in 2047. The age-standardized mortality rate is anticipated to peak at 1.69 (95% UI: 1.54, 1.69) in 2032, and the age-standardized DALYs are estimated to reach a maximum of 36.94 (95% UI: 35.17, 38.71) in 2025 (Figure 5; Supplementary Table 4). Furthermore, the total number of incident cases, prevalent cases, deaths, and DALYs among adults aged 45 and older is expected to continue increasing in the future.

### 3.8 Risk factors for NALC-related mortality

**Figure 6:**
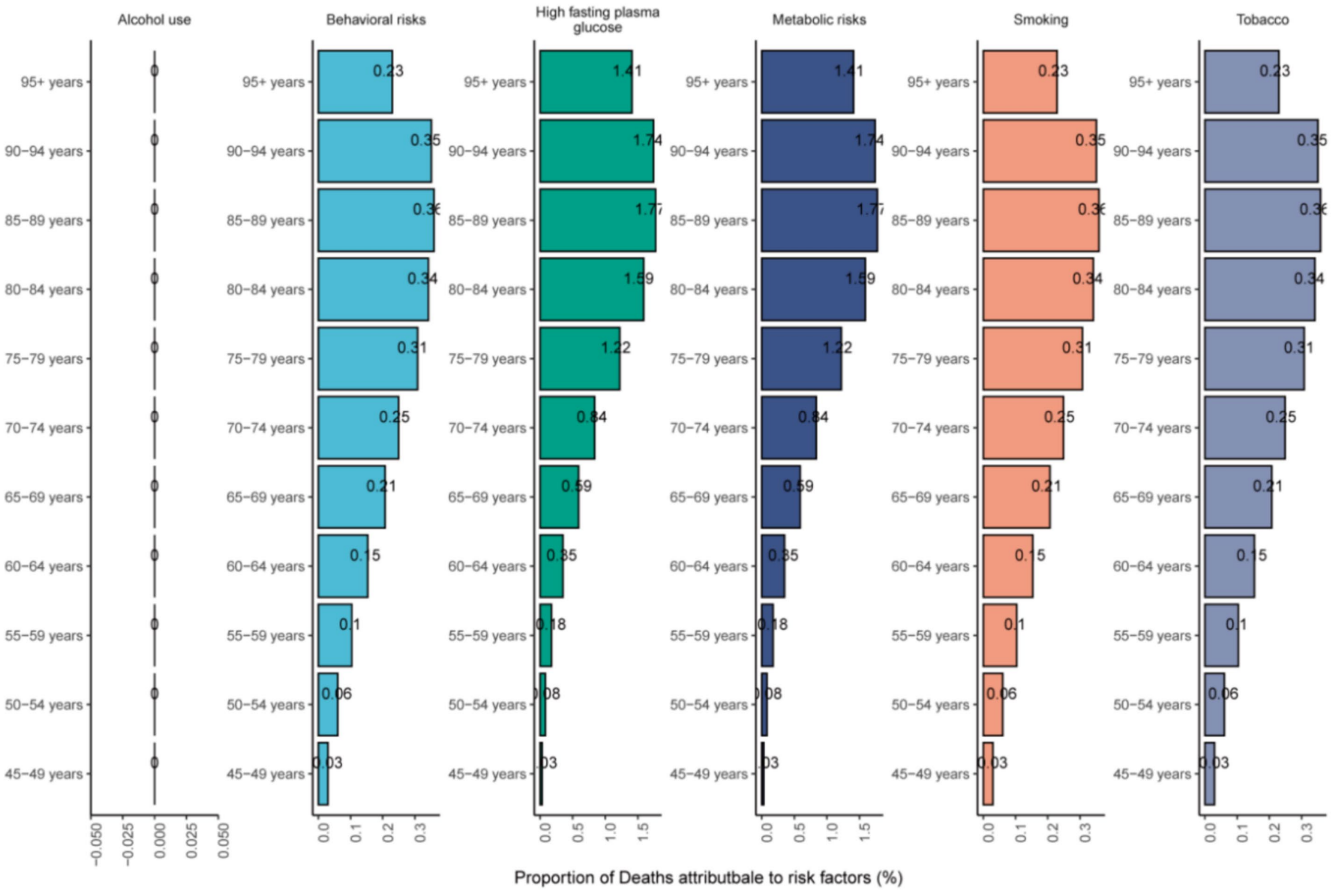
The mortality risk factors of NALC in adults 45 years and above in different age groups in 2021.

Figure 6 illustrated the proportion of NALC-related deaths attributable to different risk factors among adults aged 45 and older in 2021, stratified by age groups. Globally, high fasting plasma glucose and metabolic risks were the leading contributors across all age groups. From 1990 to 2021, the contribution of high fasting plasma glucose and metabolic risks to NALC-related deaths showed a consistent upward trend (Supplementary Figure 2). Across age groups, the contribution of metabolic risk factors to NALC mortality increased with advancing age.

## 4 Discussion

In recent years, NAFLD has progressively emerged as one of the most prevalent chronic liver conditions worldwide. NASH may progress to life-threatening complications including cirrhosis and NALC without timely and appropriate intervention, imposing a substantial public health burden globally (5, 24). This investigation focused on adults aged over 45 years as a critical demographic group, systematically analyzing the disease burden and associated risk factors of NALC across global, regional, and national levels from 1990 to 2021. Through frontier analysis, we further examined the relationship between disease burden and socio-demographic development, evaluating unrealized health potential across nations relative to their developmental benchmarks to facilitate the optimization of health resource allocation. Finally, we projected future trends in disease progression over the next three decades, providing prospective insights to inform targeted prevention and control strategies.

In 2021, the age-standardized incidence rate of NALC among adults aged 45 years and older reached 1.69 per 100, 000 population, with a prevalence rate of 2.01 per 100, 000 and a mortality rate of 1.66 per 100, 000. The DALYs stood at 36.69 per 100, 000 person-years, demonstrating a significant upward trend compared to 1990 levels, which aligns with findings from earlier studies (19, 25, 26). These results indicated that NALC has emerged as a critical health threat to middle-aged and elderly populations, with minimal gender disparities in disease burden (sex-specific differences in incidence and mortality rates being less than 5%). The disease progression exhibited a distinct age-gradient pattern, with the highest DALYs observed in the 80–84 age group. Global drivers of this trend may include population growth, diagnostic advancements, improved dietary quality, and lifestyle modifications (27). Notably, our study uncovered significant geographical variations in the distribution of this disease. The incidence rate in sub-Saharan Africa (3.81 per 100, 000) was 19 times higher than that in high-income Asia-Pacific regions (0.20 per 100, 000), consistent with findings reported by Huang et al. (28). This pattern suggested complex interrelationships between disease burden and socioeconomic development. For instance, the Age-standardized incidence rate among middle-aged and elderly populations in sub-Saharan Africa (3.81 per 100, 000) was 19-fold greater than that in high-income Asia-Pacific regions (0.20 per 100, 000). In 2021, the Islamic Republic of Mauritania, the Republic of Mozambique, and the State of Qatar exhibited the highest age-standardized incidence, prevalence, and mortality rates for NALC, respectively.

Joinpoint regression analysis revealed a significant upward trend in the burden of NALC among adults aged 45 and older globally over recent decades. Notably, the rate of increase in prevalence outpaced that of incidence and mortality, suggesting that prolonged disease duration and population aging have led to a growing number of patients experiencing long-term impacts of NALC. Importantly, we identified a nonlinear relationship between the SDI and NALC burden in middle-aged and older adults. The burden was lowest when SDI approached 0.70, but increased substantially in both low-SDI (<0.5) and high-SDI (>0.8) regions. Additionally, approximately 80% of NALC cases occurred in low- and middle-resource countries, underscoring the global disparity in disease distribution (27). Of particular concern was the sharp rise in NALC burden from 1990 to 2021 in developed regions such as the United Kingdom, Australia, and Taiwan (Province of China). These trends suggested that NALC has evolved into a global health challenge that transcends traditional boundaries between high- and low-income countries, largely driven by changing lifestyles and economic development. This dual burden reflected the complex dynamics of health transitions across the SDI spectrum. Significant disparities persisted in demographic structures, healthcare infrastructure, economic costs, clinical practices, and access to quality screening and care (29). In low-SDI countries, middle-aged and older adults faced the combined threats of infectious diseases (e.g., viral hepatitis) and metabolic liver disorders. Limited financial resources often restrict access to early imaging techniques (e.g., abdominal ultrasound), leading to late-stage diagnoses and poorer outcomes due to underdeveloped healthcare systems (30, 31). Conversely, high-SDI regions experience rising NALC burdens fueled by long-term exposure to high-calorie diets and sedentary lifestyles, which increase the risk of metabolic conditions such as NAFLD (32, 33). For instance, diabetes prevalence exceeded 20% among older adults in high-income regions, where cutting-edge medical technologies coexist with high rates of geriatric obesity and insulin resistance—factors that accelerate the progression of metabolic liver disease (34, 35). A study in Germany (a high-SDI country) demonstrated that misdiagnosis of NAFLD significantly increased the burden of comorbidities and healthcare costs, with total medical expenditures for NASH-related cirrhosis and NALC surpassing those of non-progressive cases (36). Meanwhile, Ghana (a low-middle SDI country) struggled with severe liver disease burdens due to overcrowded hospitals, prolonged wait times, and a lack of modern medical equipment (30). To tackle this complex global health issue, reimbursement policies and insurance systems must be restructured to support patient-centered, multidisciplinary care for comorbid conditions. Simultaneously, health education and promotion initiatives should raise public awareness, and interdisciplinary collaboration is essential to shift the focus of liver disease control from treatment to primary prevention (37).

Frontier analysis of middle-aged and older populations revealed that countries such as Mongolia and Mauritania exhibit actual disease burdens far exceeding SDI-predicted values, with the core challenge lying in the inability of healthcare systems to meet the demand for chronic disease management in this demographic. In Mongolia, for instance, the average fasting plasma glucose (FPG) level rose to 5.9 mmol/L in 2019, with 49.4% of individuals aged 15–69 classified as obese or overweight. HCC accounts for 39.1% of all cancer cases in Mongolia, while the average cost of liver transplantation reached $39, 589, reflecting high treatment costs and severe gaps in healthcare access (31). Despite government-subsidized screening and treatment programs, insufficient basic care services perpetuate the high burden of NALC (38). In contrast, South Korea implements HCC surveillance through its National Cancer Screening Program, mandated biennial health examinations under a universal mandatory national health insurance system, and employed machine learning models to predict HCC risk (39). These experiences highlighted that precision prevention and control for middle-aged and older adults required integrating multi-tiered healthcare resources and strengthening lifecycle management of metabolic risks. Concurrently, cost-effective screening tools and routine monitoring protocols must be developed. To date, insufficient evidence supported surveillance for NAFLD patients without cirrhosis, as ultrasound suffered from excessive missed diagnoses of early-stage cases and operator dependency, while no consensus existed on MRI-based screening for NALC (40). However, studies have shown that ultrasound screening was cost-effective for adults aged 45–64 years (41). While personalized medicine and individualized risk factor control represent the ideal approach, these methods are complex and costly, making them unsuitable for low-SDI regions (42). For low-SDI regions, we recommend adopting alternative strategies. Simplified biochemical liver marker testing, such as FibroScan-Aspartate Aminotransferase (FAST) and Fibrosis-4 (FIB-4), can effectively replace liver biopsy as diagnostic tools for high-risk NASH (43). Among these, FIB-4 demonstrated relatively high diagnostic accuracy for fibrosis stage F3 or higher (44). Indices like the Fatty Liver Index (FLI) and Hepatic Steatosis Index (HSI) can be used for the initial screening of NASH. These indices require only basic anthropometric measurements (BMI, waist circumference) and routine blood markers (liver enzymes, triglycerides), eliminating the need for expensive equipment such as ultrasound or MRI, thus making them suitable for resource-limited settings lacking imaging capabilities (45). With recent advancements in artificial intelligence (AI) technologies, AI-driven approaches could enhance disease risk diagnosis and prognosis prediction (46).

Projections indicated that the incidence of NALC in middle-aged and older populations would peak in 2042, while prevalence was expected to rise continuously until 2050. NAFLD would become increasingly prevalent in this demographic (47). Aging was a critical driver of NASH progression and played a pivotal role in NALC pathogenesis (14). The accumulation of senescent cells contributed to immune evasion, inflammatory responses, and hepatic metabolic dysregulation. The immune system was essential for clearing senescent cells, and dysregulated immune checkpoints may play a key role in advancing NASH (48). These trends were closely linked to global population aging: the proportion of individuals aged 65 and older was projected to reach 16% by 2050 globally, exceeding 25% in high-SDI regions (49). Concurrently, novel therapeutics were under active investigation. Studies demonstrated that glucagon-like peptide-1 receptor agonists (GLP-1RAs) could reverse steatohepatitis, reduce cardiovascular risk, and improve NASH histology without worsening fibrosis in middle-aged patients (50). Thyroid hormone receptor-beta (THR-*β*) agonists, which lower triglycerides and cholesterol while conferring hepatic benefits, had emerged as a primary strategy for addressing dyslipidemia, obesity, and hepatic steatosis (51). In 2024, the U.S. FDA approved Resmetirom, the first drug specifically indicated for non-cirrhotic NASH with moderate-to-severe liver fibrosis (52). Beyond emerging NASH therapies, novel approaches like targeted immunotherapy offered hope for advanced NALC patients (53). The combination of atezolizumab and bevacizumab has been approved for advanced NALC (54). However, its efficacy and safety in older adults require further evidence-based validation, necessitating careful clinical risk–benefit assessments by physicians.

The driving role of metabolic risks in NALC among middle-aged and elderly populations has been validated in this study. In 2021, elevated fasting blood glucose and metabolic risks contributed to 52% of NALC-related deaths, with the attributable fraction peaking in the 55–70 age group. This underscored their critical impact on both NALC development and mortality outcomes. An increasing number of epidemiological studies have demonstrated that diabetes is a risk factor for NASH, which is one of the causes of NALC (55). Moreover, diabetes has been identified as an independent risk factor for NALC in patients with NASH. Both diabetes and obesity are associated with an increased risk of NALC, with age being one of the significant risk factors (56). This was because hyperglycemia, insulin resistance, and hyperinsulinemia can disrupt liver homeostasis, thereby contributing to the development of NALC (57). Epidemiological evidence also demonstrated that NALC development was associated with multiple metabolic syndrome manifestations and lifestyle factors, including obesity, advanced age, dysregulated lipid metabolism, insulin resistance, dietary patterns, sedentary behavior, chronic stress, hypertension, and persistent inflammation (10, 47, 58). Although NALC exhibited multifactorial etiology, most pathways converged on inflammatory mechanisms (59). Hyperinsulinemia promotes hepatic *de novo* lipogenesis, leading to the accumulation of newly synthesized lipids in the liver and exacerbating NAFLD (60). Both NAFLD and diabetes are closely linked to obesity, insulin resistance, heightened inflammation, and increased oxidative stress. These factors collectively drive the development and progression of NALC by promoting cellular growth and proliferation, inhibiting apoptosis, and enhancing angiogenesis. Additionally, insulin resistance and hyperinsulinemia stimulate increased secretion of insulin-like growth factor I (IGF-I), which further promotes hepatic cell proliferation and suppresses apoptosis, thereby augmenting the risk of NALC (57). Molecular investigations revealed that lipotoxicity induced DNA damage and mutations in metabolic regulatory genes (e.g., FOXO1, a tumor suppressor), thereby compromising cellular repair capacity (61). Chronic activation of Toll-like receptors (TLRs) triggered the release of pro-inflammatory cytokines (TNF-*α*, IL-1β, IL-18) and synergized with inflammasomes to amplify inflammatory cascades, culminating in hepatocyte injury, fibrosis, and immunosuppression—processed that collectively promote malignant transformation (62, 63). Notably, this risk exhibited a “temporal window” effect: A 7–10% weight loss in middle-aged populations (45–65 years) significantly ameliorated NASH-related fibrosis and indirectly reduces NALC risk, with ≥10% weight reduction achieving histological improvement in 90% of NASH patients (64). Concurrently, the preserved hepatic regenerative capacity in middle age allowed metabolic interventions (e.g., weight loss, insulin sensitization) to reverse lipotoxicity, inflammation, and fibrotic remodeling (2). Conversely, elderly populations exhibited diminished risk mitigation due to accumulated senescent cells (65). Therefore, there was an urgent need to implement lifestyle modifications in middle-aged populations through targeted metabolic interventions, including weight reduction, tailored low-calorie dietary regimens, and enhanced physical activity, along with comprehensive metabolic control targeting blood glucose, blood pressure, lipid profile, and body weight, which may potentially mitigate the future disease burden of NALC (66).

This study has several limitations that warrant consideration. First, our findings are derived from the GBD database, where data heterogeneity across countries and regions—particularly in low and middle-low SDI settings—may affect result accuracy due to potential under-reporting, misdiagnosis, and incomplete cancer registry systems. Second, the analysis specifically addressed NALC attributable to NASH without stratification by precise pathological subtypes. Third, our temporal projections assumed static sociodemographic trends and did not account for the impacts of the COVID-19 pandemic, which may introduce non-negligible biases into long-term forecasting. These limitations underscored the imperative to Chalasani et al. (1) strengthen data infrastructure in resource-limited regions, (2) implement granular disease subtyping frameworks, and (3) integrate pandemic-related demographic shocks into predictive models. Future investigations should prioritize comprehensive assessments of pandemic-induced healthcare disruptions to better capture evolving population health dynamics.

## 5 Conclusion

This study investigated the disease burden of NALC among adults aged 45 years and older from 1990 to 2021. The findings reveal a persistent global increase in NALC burden within this population, with marked variations across SDI regions and individual nations. Current projections indicate the prevalence trend will continue its upward trajectory. Notably, fasting plasma glucose and metabolic risk factors were identified as significant contributors to NALC pathogenesis. These findings suggested the urgent need for developing targeted strategies addressing metabolic-related risk factors and implementing standardized screening protocols to mitigate the growing disease burden of NALC in aging populations.

## Data Availability

The original contributions presented in the study are included in the article/Supplementary material, further inquiries can be directed to the corresponding author.

## Footnotes

^1^ https://vizhub.healthdata.org/gbd-results/
