# Supplementary material for: Global, regional, and national burden of NASH related liver cancer in adults aged 45 and above: an analysis from the GBD 2021 and forecast to 2050
Source: Front Nutr. 2025 Aug 19;12:1651357. doi: 10.3389/fnut.2025.1651357 (PMC12401703; doi:10.3389/fnut.2025.1651357)
Supplement: Supplementary file 1 [file Table_1.DOCX]

Supplementary Material

# Supplementary Figures and Tables

## Supplementary Figures


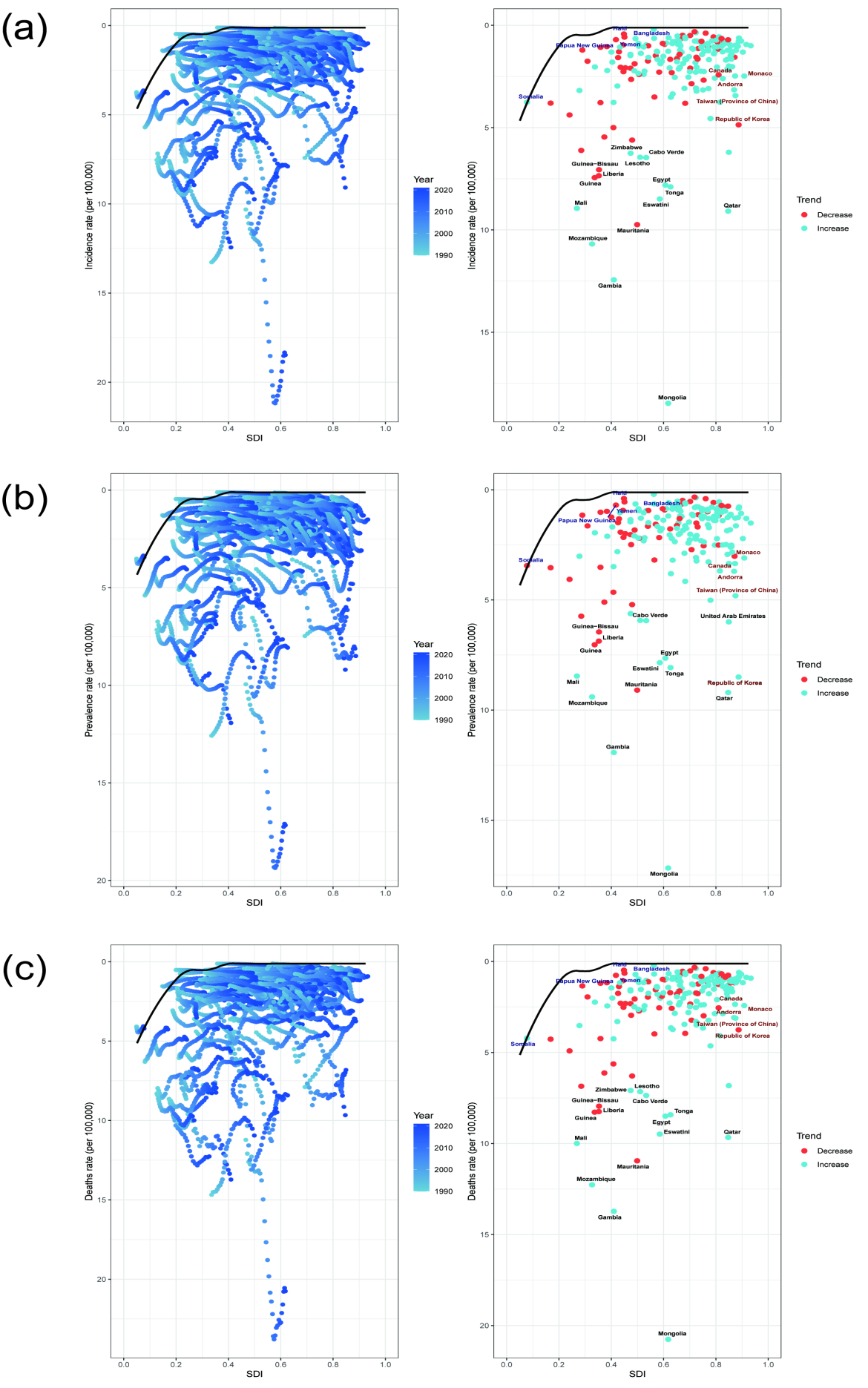


**Supplementary Figure 1.** Frontier analysis of age-standardized incidence rate, prevalence rate, and mortality rate of NALC.


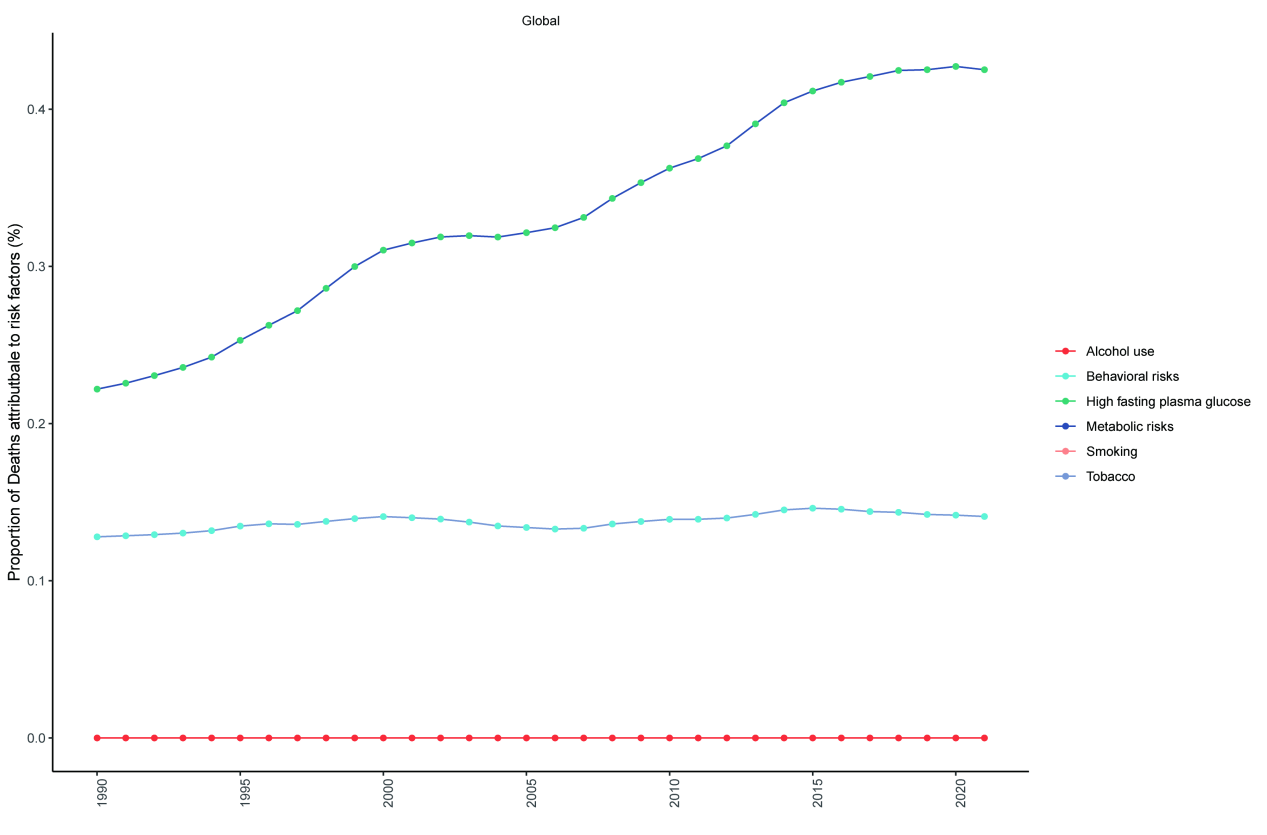


**Supplementary Figure 2.** Trends in risk factors for mortality, 1990-2021.

## Supplementary Tables

**Supplementary Table 1.** Global number of NALC cases by gender among adults aged 45 and above from 1990 to 2021.

| **Location** | **Sex** | **Incidence** | | **Prevalence** | | **Deaths** | | **DALYs** | |
| --- | --- | --- | --- | --- | --- | --- | --- | --- | --- |
|  |  | 1990 (95%UI) | 2021 (95%UI) | 1990 (95%UI) | 2021 (95%UI) | 1990 (95%UI) | 2021 (95%UI) | 1990 (95%UI) | 2021 (95%UI) |
| Global | Male | 6384.56(4346.42,8991.63) | 20412.94(13980.65,28639.64) | 7135.17(4884.55,9988.17) | 25644.38(17579.47,35901.25) | 6453.69(4376.90,9117.30) | 19375.91(13218.43,27183.80) | 169004.86(114259.76,238974.31) | 461253.83(313352.93,650005.76) |
|  | Female | 6576.15(4468.95,9218.23) | 19308.00(13465.02,26263.86) | 6867.44(4707.48,9569.27) | 22147.08(15430.42,30352.20) | 6914.07(4670.03,9731.14) | 19419.88(13539.34,26461.13) | 162065.14(109827.62,228394.69) | 416099.41(291670.12,565933.55) |
| **SDI** | | | | | | | | | |
| High SDI | Male | 1711.70(1188.76,2410.17) | 5759.09(3959.32,8064.45) | 2201.85(1551.33,3049.56) | 9033.18(6164.59,12661.46) | 1582.09(1084.68,2259.18) | 4809.48(3295.48,6762.72) | 38580.67(26366.43,55304.99) | 99602.36(68118.57,140269.94) |
|  | Female | 1668.71(1142.41,2350.74) | 5068.10(3350.29,7152.27) | 1956.03(1354.86,2716.77) | 7044.47(4608.70,10035.00) | 1660.83(1124.11,2357.16) | 4625.36(3045.73,6591.93) | 33703.72(22793.47,47906.10) | 82076.02(54947.00,115971.63) |
| High-middle SDI | Male | 1371.81(934.28,1919.07) | 3887.69(2559.16,5584.01) | 1479.20(1006.55,2069.64) | 4730.81(3082.35,6798.52) | 1410.61(959.84,1972.34) | 3696.05(2442.36,5311.91) | 37556.68(25408.19,52668.95) | 88471.02(58062.04,127751.35) |
|  | Female | 1309.67(907.97,1796.67) | 3399.27(2349.26,4725.67) | 1317.83(912.64,1807.72) | 3766.27(2610.08,5230.49) | 1401.81(971.81,1922.05) | 3449.92(2385.71,4783.39) | 32742.49(22677.94,44901.04) | 72220.30(50196.14,100205.43) |
| Middle SDI | Male | 1892.93(1266.21,2694.28) | 6803.89(4498.11,9706.50) | 2006.98(1340.09,2856.02) | 7775.47(5109.34,11167.41) | 1973.02(1319.96,2815.40) | 6673.75(4434.10,9498.55) | 53208.00(35478.90,75918.86) | 165166.06(108978.50,236392.35) |
|  | Female | 1954.22(1352.31,2689.11) | 6073.46(4290.98,8322.03) | 1973.55(1368.38,2718.02) | 6612.15(4643.42,9085.04) | 2084.95(1439.46,2868.34) | 6198.46(4389.91,8467.67) | 51679.19(35904.82,71063.13) | 138292.23(98011.20,188668.24) |
| Low-middle SDI | Male | 853.64(544.96,1272.35) | 2884.84(1898.47,4195.98) | 885.14(564.71,1323.13) | 3002.68(1968.80,4384.09) | 899.01(575.48,1341.89) | 3048.58(2007.03,4441.09) | 24182.07(15455.94,36336.55) | 78376.08(51212.42,114928.57) |
|  | Female | 950.57(583.33,1539.50) | 3157.71(2155.91,4379.52) | 942.66(581.25,1520.40) | 3144.53(2137.07,4370.05) | 1020.20(624.68,1658.10) | 3399.82(2326.72,4699.91) | 25422.70(15719.37,40838.37) | 81526.41(55371.03,113030.20) |
| Low SDI | Male | 549.77(293.21,939.32) | 1065.13(601.31,1756.87) | 557.18(297.58,952.77) | 1088.60(612.55,1802.80) | 583.94(311.01,997.89) | 1135.31(640.62,1871.64) | 15354.97(8193.89,26257.61) | 29340.20(16363.90,48835.24) |
|  | Female | 686.48(369.89,1216.24) | 1596.73(974.48,2442.29) | 671.08(364.79,1185.15) | 1566.60(955.01,2403.18) | 739.14(395.12,1317.86) | 1732.53(1059.51,2650.63) | 18360.46(9998.38,32448.01) | 41698.91(25383.43,64174.66) |
| **GBD 21 regions** | | | | | | | | | |
| Andean Latin America | Male | 10.73(5.49,18.93) | 46.32(23.12,82.06) | 10.37(5.28,18.32) | 44.38(22.02,79.29) | 11.91(6.12,20.97) | 51.79(25.95,91.69) | 265.15(134.91,467.69) | 1055.14(518.68,1889.92) |
|  | Female | 35.93(19.25,59.17) | 135.88(73.03,222.82) | 34.19(18.26,56.40) | 128.79(68.82,211.80) | 40.00(21.59,65.97) | 152.46(81.99,249.77) | 875.26(469.68,1448.00) | 3089.75(1642.81,5100.68) |
| Australasia | Male | 18.11(10.24,29.85) | 173.87(97.48,284.92) | 20.81(11.82,33.98) | 263.32(146.65,433.80) | 18.05(10.18,29.93) | 151.77(84.35,247.25) | 420.89(235.48,697.51) | 3215.93(1783.42,5225.56) |
|  | Female | 15.35(9.07,24.04) | 136.59(78.06,219.44) | 16.05(9.57,25.13) | 159.39(92.31,252.32) | 15.85(9.22,24.96) | 138.09(77.79,224.82) | 335.45(195.43,528.12) | 2637.17(1494.92,4269.25) |
| Caribbean | Male | 16.80(8.87,28.54) | 48.53(26.23,81.00) | 16.47(8.64,27.96) | 49.99(26.90,83.90) | 18.41(9.73,31.28) | 52.05(28.07,86.91) | 408.33(213.15,695.73) | 1159.56(619.79,1953.53) |
|  | Female | 28.74(16.28,46.22) | 58.29(32.72,94.72) | 27.74(15.70,44.50) | 57.43(32.34,92.87) | 31.73(17.97,51.15) | 64.38(35.88,104.61) | 683.31(386.41,1094.28) | 1343.31(751.61,2172.19) |
| Central Asia | Male | 95.77(51.93,160.74) | 178.80(95.53,303.64) | 99.77(53.94,167.46) | 187.11(99.47,318.33) | 100.69(54.75,169.33) | 188.18(100.53,318.61) | 2701.81(1461.38,4537.97) | 4953.32(2621.37,8397.96) |
|  | Female | 117.77(65.12,192.76) | 236.20(132.38,388.47) | 115.72(63.87,189.20) | 233.10(130.26,382.37) | 128.20(71.23,210.46) | 256.89(143.94,421.61) | 3032.24(1677.72,4960.91) | 6023.66(3355.28,9851.48) |
| Central Europe | Male | 133.40(74.38,219.72) | 268.97(160.81,422.96) | 132.98(73.93,219.88) | 272.70(162.00,429.41) | 143.59(80.50,235.82) | 289.05(173.05,455.25) | 3411.90(1903.18,5643.50) | 6246.22(3709.01,9860.43) |
|  | Female | 218.41(130.04,345.07) | 329.88(205.14,493.31) | 206.07(122.24,325.95) | 313.73(194.14,471.04) | 243.17(145.53,383.73) | 370.75(231.37,554.07) | 5133.30(3051.06,8136.56) | 6957.10(4322.97,10444.61) |
| Central Latin America | Male | 63.47(39.76,95.78) | 289.15(188.65,416.75) | 62.61(39.18,94.64) | 287.51(186.83,414.20) | 69.46(43.57,104.78) | 316.13(206.38,454.65) | 1602.32(1002.25,2423.33) | 6943.66(4502.20,9987.17) |
|  | Female | 109.71(69.56,162.09) | 403.22(267.03,578.66) | 107.91(68.30,159.49) | 394.07(260.88,563.55) | 119.83(76.06,177.20) | 445.49(294.95,637.68) | 2779.50(1759.47,4108.33) | 9547.85(6316.73,13605.66) |
| Central Sub-Saharan Africa | Male | 43.92(14.29,107.63) | 78.81(26.06,195.88) | 45.18(14.64,110.51) | 83.79(27.63,208.45) | 45.93(14.93,111.91) | 82.22(26.96,208.61) | 1282.10(411.80,3105.64) | 2317.99(752.81,5905.44) |
|  | Female | 51.88(15.78,140.20) | 111.56(32.65,323.27) | 52.74(16.13,141.70) | 113.55(33.33,328.31) | 54.77(16.55,149.12) | 119.37(34.66,355.27) | 1473.53(450.73,3975.82) | 3117.46(908.34,9225.57) |
| East Asia | Male | 1851.87(1232.81,2631.91) | 5971.34(3732.95,8997.95) | 2010.42(1334.88,2860.94) | 7437.53(4630.84,11268.26) | 1895.58(1262.00,2693.43) | 5434.05(3413.40,8161.74) | 52720.44(34837.04,75109.43) | 132635.24(82578.06,200723.72) |
|  | Female | 1744.40(1195.39,2402.74) | 5204.79(3532.25,7380.35) | 1793.80(1227.75,2472.75) | 6127.41(4159.08,8727.73) | 1834.38(1257.98,2521.07) | 5007.88(3402.71,7117.18) | 46815.58(32028.25,64466.80) | 109937.64(74660.35,157050.67) |
| Eastern Europe | Male | 147.56(105.68,198.57) | 331.55(237.07,446.44) | 153.67(109.86,207.50) | 344.35(245.43,465.20) | 154.48(110.90,207.78) | 349.30(250.60,469.87) | 4035.04(2889.79,5440.55) | 8454.61(6036.43,11413.37) |
|  | Female | 194.71(142.12,257.98) | 374.35(270.45,495.79) | 189.39(138.27,250.99) | 353.34(255.29,468.10) | 212.52(155.03,281.99) | 420.52(304.07,556.85) | 4753.45(3470.36,6310.99) | 8211.06(5932.40,10870.60) |
| Easten Sub-Saharan Africa | Male | 166.83(84.95,304.87) | 379.22(190.04,697.54) | 167.59(85.24,307.50) | 390.13(195.34,716.66) | 176.98(89.97,323.32) | 400.38(201.00,734.72) | 4650.81(2357.94,8542.02) | 10690.18(5321.14,19679.29) |
|  | Female | 278.53(165.74,441.05) | 657.81(395.68,1005.14) | 272.52(162.99,430.25) | 644.58(388.34,981.37) | 299.50(177.26,475.54) | 714.71(430.30,1094.02) | 7487.22(4482.93,11798.38) | 17170.69(10360.17,26165.58) |
| High-income Asia Pacific | Male | 818.38(576.80,1161.18) | 1532.57(1000.55,2285.66) | 1167.67(832.44,1611.96) | 2872.56(1851.46,4316.11) | 720.98(500.13,1040.64) | 1191.45(788.86,1767.03) | 19151.60(13235.17,27770.35) | 22600.07(14558.19,34338.10) |
|  | Female | 724.47(481.16,1060.99) | 1601.56(967.26,2412.35) | 970.87(660.97,1370.01) | 2714.59(1594.47,4219.73) | 669.85(437.52,1003.95) | 1281.53(772.93,1922.74) | 14727.86(9499.76,22320.10) | 19659.88(11935.16,29711.14) |
| High-income North America | Male | 387.20(287.51,504.59) | 2187.48(1597.96,2849.95) | 467.43(347.36,608.71) | 3204.19(2340.78,4172.22) | 347.16(258.20,452.98) | 1767.18(1291.40,2307.64) | 7509.83(5569.91,9807.10) | 38274.05(27981.41,50018.78) |
|  | Female | 373.84(279.71,478.51) | 1619.45(1170.21,2132.00) | 395.79(296.65,506.72) | 1950.77(1413.99,2561.07) | 370.68(276.47,477.91) | 1481.83(1060.79,1963.05) | 6935.87(5215.33,8908.24) | 28185.13(20540.23,36960.52) |
| North Africa and Middle East | Male | 266.66(140.41,471.43) | 1420.94(797.14,2358.48) | 273.88(143.88,484.82) | 1510.48(840.55,2519.94) | 283.36(149.29,500.42) | 1485.22(830.61,2447.16) | 7271.31(3804.54,12891.58) | 37406.98(20550.43,62154.69) |
|  | Female | 322.80(149.93,675.28) | 1207.84(695.77,1899.14) | 322.58(150.76,668.65) | 1247.07(716.88,1961.05) | 346.85(160.42,727.31) | 1278.91(735.95,2019.47) | 8602.19(4005.19,17758.27) | 31152.40(17701.44,49264.53) |
| Oceania | Male | 4.85(2.01,11.85) | 11.42(4.78,25.57) | 5.07(2.09,12.41) | 12.20(5.09,27.49) | 5.09(2.11,12.44) | 11.89(4.97,26.86) | 139.15(57.04,341.95) | 323.48(133.41,741.66) |
|  | Female | 4.40(1.72,10.39) | 10.37(4.93,20.08) | 4.57(1.79,10.80) | 10.99(5.20,21.39) | 4.60(1.80,10.84) | 10.76(5.11,20.90) | 125.53(48.75,296.82) | 287.48(133.85,571.84) |
| South Asia | Male | 665.81(468.99,912.92) | 2411.65(1719.21,3281.02) | 694.79(488.73,954.41) | 2488.64(1769.53,3395.21) | 698.39(492.87,958.13) | 2563.89(1828.88,3487.03) | 19016.56(13386.39,26176.93) | 64361.00(45676.16,88060.02) |
|  | Female | 501.57(355.82,665.00) | 2299.16(1673.58,2997.13) | 499.80(355.33,662.26) | 2260.46(1641.94,2949.99) | 535.06(379.92,709.28) | 2494.70(1815.93,3247.06) | 13607.66(9693.06,17999.12) | 58353.11(42284.46,76210.31) |
| Southeast Asia | Male | 663.18(379.93,1073.06) | 2152.23(1214.60,3565.31) | 699.20(400.53,1132.16) | 2377.70(1337.04,3953.64) | 696.77(400.37,1122.72) | 2209.80(1246.12,3665.57) | 18789.16(10816.16,30382.37) | 58165.46(32832.09,96915.13) |
|  | Female | 601.80(363.23,933.54) | 1725.13(925.18,2773.24) | 608.49(368.46,942.91) | 1839.85(989.50,2978.56) | 643.61(388.37,1000.57) | 1798.47(964.37,2867.49) | 15911.24(9672.83,24605.03) | 41177.02(21771.99,65485.97) |
| Southern Latin America | Male | 13.26(6.81,23.14) | 77.59(41.42,129.46) | 13.25(6.80,23.20) | 80.35(42.77,133.85) | 14.28(7.36,24.83) | 82.04(43.79,136.88) | 335.02(172.25,585.65) | 1780.70(945.05,2977.30) |
|  | Female | 15.30(8.29,25.05) | 78.63(42.54,128.28) | 14.76(7.99,24.10) | 78.85(42.70,128.61) | 16.88(9.15,27.57) | 85.61(45.98,140.29) | 361.00(195.17,587.96) | 1739.23(937.82,2826.24) |
| Southern Sub-Saharan Africa | Male | 55.25(20.03,109.92) | 257.39(168.10,382.26) | 55.89(20.18,111.21) | 257.89(167.64,383.77) | 59.25(21.71,118.14) | 275.16(180.15,407.87) | 1498.88(543.05,2976.61) | 6926.43(4470.56,10362.20) |
|  | Female | 78.94(44.34,134.37) | 247.91(165.08,364.34) | 76.81(43.29,130.55) | 239.08(159.14,351.21) | 86.75(48.78,147.90) | 271.46(181.22,399.22) | 2010.73(1137.22,3414.18) | 6331.07(4200.64,9317.49) |
| Tropical Latin America | Male | 35.99(26.27,47.50) | 165.10(119.53,219.47) | 36.44(26.56,48.19) | 169.83(122.93,225.81) | 38.61(28.27,50.89) | 176.91(128.16,235.29) | 959.93(700.61,1269.04) | 4194.19(3035.75,5579.88) |
|  | Female | 58.78(43.63,76.57) | 203.94(147.80,267.33) | 58.07(43.15,75.64) | 199.35(145.19,260.11) | 64.06(47.57,83.48) | 225.73(163.61,295.93) | 1510.18(1124.78,1966.15) | 4875.78(3566.89,6341.65) |
| Western Europe | Male | 547.50(339.37,837.48) | 1743.31(1047.84,2719.83) | 619.28(384.51,944.63) | 2612.98(1562.30,4048.17) | 550.71(342.31,842.44) | 1559.75(937.29,2424.28) | 12377.44(7654.02,18977.11) | 30871.49(18448.70,48070.53) |
|  | Female | 587.62(373.56,882.26) | 1524.05(926.27,2327.40) | 608.61(386.22,914.40) | 1963.42(1187.13,3006.31) | 638.63(403.85,964.94) | 1560.48(948.34,2390.96) | 11756.22(7456.46,17638.84) | 26783.97(16389.41,40564.58) |
| Western Sub-Saharan Africa | Male | 378.03(177.57,676.19) | 686.71(384.31,1148.37) | 382.42(179.45,684.92) | 696.74(387.52,1170.97) | 404.02(190.80,724.55) | 737.70(413.82,1230.29) | 10457.17(4923.54,18799.00) | 18678.13(10336.53,31433.88) |
|  | Female | 511.19(242.91,1016.82) | 1141.39(722.18,1680.01) | 490.97(233.67,976.85) | 1117.27(704.21,1650.74) | 557.12(265.80,1107.70) | 1239.88(789.67,1825.38) | 13147.81(6268.13,26203.44) | 29518.65(18589.58,43778.10) |

**Supplementary Table 2.** Age-standardized incidence, prevalence, mortality rates, and DALYs of NALC by age group among adults aged 45 and above in 2021.

| **Location** | **Age (years)** | **Incidence (per 100,000 population, 95%UI)** | **Prevalence (per 100,000 population, 95%UI)** | **Deaths (per 100,000 population, 95%UI)** | **DALYs (per 100,000 population, 95%UI)** |
| --- | --- | --- | --- | --- | --- |
| Global | 45-49 | 0.36(0.26,0.50) | 0.55(0.39,0.75) | 0.30(0.21,0.41) | 12.93(8.95,17.89) |
|  | 50-54 | 0.67(0.46,0.92) | 0.99(0.69,1.36) | 0.55(0.38,0.76) | 21.32(14.74,29.36) |
|  | 55-59 | 1.07(0.74,1.54) | 1.50(1.04,2.15) | 0.94(0.65,1.36) | 31.71(21.90,46.04) |
|  | 60-64 | 1.65(1.16,2.34) | 2.18(1.55,3.09) | 1.49(1.05,2.11) | 43.41(30.51,61.34) |
|  | 65-69 | 2.32(1.62,3.13) | 2.84(1.99,3.83) | 2.19(1.53,2.96) | 53.84(37.67,72.48) |
|  | 70-74 | 2.92(2.05,3.85) | 3.31(2.34,4.39) | 2.87(2.02,3.80) | 58.16(40.80,76.91) |
|  | 75-79 | 3.90(2.81,5.28) | 4.21(3.03,5.62) | 4.09(2.94,5.53) | 66.14(47.54,89.59) |
|  | 80-84 | 4.77(3.33,6.54) | 4.90(3.39,6.81) | 5.35(3.74,7.30) | 67.75(47.37,92.61) |
|  | 85-89 | 5.84(4.00,7.95) | 5.90(4.01,8.10) | 6.19(4.31,8.42) | 62.55(43.43,84.94) |
|  | 90-94 | 4.90(3.14,7.01) | 3.34(2.13,4.78) | 6.80(4.38,9.73) | 59.63(38.47,85.26) |
|  | 95+ | 4.03(2.24,6.49) | 2.07(1.15,3.33) | 6.26(3.47,10.13) | 51.03(28.37,82.81) |
| **SDI** | | | | | |
| High SDI | 45-49 | 0.27(0.18,0.38) | 0.59(0.39,0.82) | 0.17(0.12,0.24) | 7.48(5.04,10.57) |
|  | 50-54 | 0.56(0.77,0.39) | 1.14(0.79,1.54) | 0.37(0.25,0.50) | 14.19(9.79,19.5) |
|  | 55-59 | 1.06(1.54,0.72) | 1.91(1.30,2.82) | 0.76(0.52,1.10) | 25.68(17.45,37.41) |
|  | 60-64 | 1.80(2.56,1.27) | 3.08(2.16,4.38) | 1.34(0.94,1.92) | 39.20(27.52,56.22) |
|  | 65-69 | 2.46(1.69,3.33) | 3.86(2.64,5.28) | 1.97(1.35,2.69) | 48.56(33.32,66.15) |
|  | 70-74 | 3.18(2.20,4.31) | 4.63(3.17,6.25) | 2.67(1.83,3.61) | 53.93(37.03,72.93) |
|  | 75-79 | 4.69(3.25,6.55) | 6.59(4.51,9.04) | 4.29(2.97,5.98) | 69.17(47.84,96.54) |
|  | 80-84 | 5.62(3.73,8.06) | 7.68(5.06,10.93) | 5.57(3.65,7.93) | 70.54(46.39,100.24) |
|  | 85-89 | 7.52(4.97,10.52) | 9.95(6.64,14.00) | 6.69(4.37,9.45) | 67.75(44.25,95.47) |
|  | 90-94 | 5.66(3.52,8.17) | 4.31(2.73,6.31) | 7.55(4.74,10.96) | 66.22(41.58,96.18) |
|  | 95+ | 4.99(2.74,8.05) | 2.58(1.41,4.16) | 7.63(4.14,12.40) | 61.76(33.48,100.51) |
| High-middle SDI | 45-49 | 0.36(0.25,0.51) | 0.55(0.39,0.80) | 0.27(0.19,0.39) | 11.82(8.38,16.90) |
|  | 50-54 | 0.60(0.42,0.83) | 0.91(0.63,1.25) | 0.47(0.33,0.65) | 18.10(12.73,25.01) |
|  | 55-59 | 0.86(0.58,1.24) | 1.22(0.82,1.75) | 0.73(0.50,1.07) | 24.72(16.81,36.12) |
|  | 60-64 | 1.30(0.90,1.88) | 1.70(1.17,2.46) | 1.16(0.81,1.67) | 33.76(23.45,48.66) |
|  | 65-69 | 1.84(1.26,2.47) | 2.21(1.51,2.96) | 1.74(1.19,2.33) | 42.63(29.13,57.20) |
|  | 70-74 | 2.20(1.50,2.95) | 2.41(1.65,3.22) | 2.19(1.50,2.93) | 44.36(30.25,59.37) |
|  | 75-79 | 2.96(2.11,3.99) | 2.96(2.11,4.01) | 3.18(2.25,4.27) | 51.29(36.35,68.99) |
|  | 80-84 | 3.62(2.55,5.00) | 3.28(2.32,4.51) | 4.21(3.00,5.81) | 53.14(37.97,73.31) |
|  | 85-89 | 4.19(2.89,5.75) | 3.36(2.32,4.60) | 4.82(3.35,6.62) | 48.60(33.79,66.70) |
|  | 90-94 | 3.68(2.42,5.31) | 2.28(1.50,3.29) | 5.25(3.46,7.56) | 46.05(30.28,66.44) |
|  | 95+ | 2.83(1.6,4.56) | 1.44(0.81,2.31) | 4.48(2.53,7.18) | 37.07(20.98,59.25) |
| Middle SDI | 45-49 | 0.42(0.30,0.59) | 0.61(0.43,0.85) | 0.35(0.25,0.48) | 14.98(10.58,20.75) |
|  | 50-54 | 0.74(0.52,1.03) | 1.06(0.74,1.47) | 0.62(0.43,0.86) | 23.89(16.67,32.89) |
|  | 55-59 | 1.13(0.77,1.61) | 1.50(1.02,2.16) | 1.01(0.70,1.44) | 34.12(23.53,48.56) |
|  | 60-64 | 1.74(1.22,2.47) | 2.13(1.48,3.03) | 1.62(1.13,2.30) | 47.15(32.86,66.76) |
|  | 65-69 | 2.41(1.66,3.20) | 2.75(1.89,3.69) | 2.34(1.62,3.13) | 57.33(39.85,76.93) |
|  | 70-74 | 3.04(2.15,4.04) | 3.14(2.20,4.19) | 3.11(2.20,4.13) | 62.87(44.45,83.55) |
|  | 75-79 | 3.90(2.80,5.29) | 3.64(2.62,4.97) | 4.28(3.10,5.80) | 69.28(50.02,93.75) |
|  | 80-84 | 5.09(3.66,6.98) | 4.31(3.11,5.93) | 6.03(4.40,8.22) | 76.35(55.73,104.21) |
|  | 85-89 | 5.98(4.20,7.95) | 4.41(3.06,5.86) | 7.14(5.09,9.53) | 71.99(51.23,96.29) |
|  | 90-94 | 5.44(3.54,7.79) | 3.28(2.12,4.71) | 7.83(5.17,11.20) | 68.66(45.31,98.17) |
|  | 95+ | 3.72(2.05,5.87) | 1.89(1.04,2.98) | 5.92(3.24,9.30) | 48.61(26.67,76.35) |
| Low-middle SDI | 45-49 | 0.33(0.22,0.46) | 0.42(0.28,0.59) | 0.31(0.21,0.43) | 13.24(8.89,18.48) |
|  | 50-54 | 0.65(0.42,0.90) | 0.82(0.53,1.14) | 0.61(0.40,0.85) | 23.49(15.28,32.65) |
|  | 55-59 | 1.15(0.77,1.67) | 1.39(0.93,2.01) | 1.13(0.75,1.64) | 38.01(25.30,55.44) |
|  | 60-64 | 1.69(1.17,2.40) | 1.86(1.29,2.65) | 1.70(1.17,2.41) | 49.45(34.23,70.16) |
|  | 65-69 | 2.44(1.66,3.37) | 2.49(1.70,3.44) | 2.55(1.75,3.53) | 62.60(42.82,86.60) |
|  | 70-74 | 2.99(2.07,4.05) | 2.73(1.89,3.71) | 3.29(2.29,4.46) | 66.66(46.27,90.28) |
|  | 75-79 | 3.51(2.44,4.83) | 2.90(2.02,3.99) | 4.11(2.86,5.64) | 66.58(46.29,91.33) |
|  | 80-84 | 3.90(2.75,5.41) | 2.84(1.99,3.94) | 4.92(3.47,6.81) | 62.56(44.09,86.76) |
|  | 85-89 | 3.61(2.55,5.14) | 2.26(1.59,3.22) | 4.82(3.42,6.82) | 48.75(34.52,69.08) |
|  | 90-94 | 3.01(1.96,4.36) | 1.73(1.13,2.51) | 4.43(2.91,6.43) | 38.83(25.51,56.36) |
|  | 95+ | 1.76(1.00,2.84) | 0.89(0.51,1.43) | 2.84(1.63,4.59) | 23.47(13.46,38.00) |
| Low SDI | 45-49 | 0.40(0.23,0.65) | 0.50(0.28,0.81) | 0.38(0.21,0.61) | 16.38(9.20,26.51) |
|  | 50-54 | 0.79(0.45,1.27) | 0.98(0.56,1.57) | 0.76(0.43,1.21) | 29.22(16.59,46.53) |
|  | 55-59 | 1.31(0.76,2.10) | 1.55(0.91,2.50) | 1.29(0.75,2.07) | 43.53(25.37,69.86) |
|  | 60-64 | 1.93(1.19,3.05) | 2.09(1.29,3.30) | 1.96(1.21,3.08) | 57.04(35.27,89.93) |
|  | 65-69 | 3.02(1.85,4.58) | 3.03(1.85,4.60) | 3.19(1.94,4.84) | 78.26(47.64,118.85) |
|  | 70-74 | 4.07(2.42,6.19) | 3.67(2.19,5.57) | 4.51(2.69,6.86) | 91.47(54.48,139.08) |
|  | 75-79 | 5.13(3.20,7.43) | 4.17(2.61,6.04) | 6.03(3.76,8.71) | 97.91(60.89,141.51) |
|  | 80-84 | 6.04(3.71,9.20) | 4.32(2.65,6.57) | 7.66(4.70,11.61) | 97.43(59.87,147.61) |
|  | 85-89 | 6.48(4.23,9.65) | 3.98(2.60,5.94) | 8.74(5.71,13.10) | 88.41(57.79,132.72) |
|  | 90-94 | 6.34(3.72,9.71) | 3.62(2.12,5.55) | 9.33(5.58,14.24) | 81.80(48.92,125.03) |
|  | 95+ | 4.54(2.22,7.66) | 2.29(1.12,3.86) | 7.31(3.60,12.27) | 61.37(30.18,102.92) |

**Supplementary Table 3.** Disease burden of NALC among adults aged 45 and above in 204 countries or regions from 1990 to 2021.

| **Location** | **Incidence** | | **Prevalence** | | **Deaths** | | **DALYs** | |
| --- | --- | --- | --- | --- | --- | --- | --- | --- |
|  | 1990 (per 100,000 population, 95%UI) | 2021 (per 100,000 population, 95%UI) | 1990 (per 100,000 population, 95%UI) | 2021 (per 100,000 population, 95%UI) | 1990 (per 100,000 population, 95%UI) | 2021 (per 100,000 population, 95%UI) | 1990 (per 100,000 population, 95%UI) | 2021 (per 100,000 population, 95%UI) |
| People's Republic of China | 3453.33(2427.35,4693.53) | 10636.27(7259.41,14850.40) | 1.56(1.10,2.13) | 2.15(1.46,3.01) | 1.68(1.18,2.27) | 1.73(1.18,2.41) | 40.37(28.30,54.81) | 38.35(26.13,53.79) |
| Democratic People's Republic of Korea | 78.27(30.96,168.84) | 135.51(60.50,266.48) | 1.79(0.70,3.87) | 1.56(0.70,3.06) | 1.91(0.74,4.21) | 1.55(0.69,3.09) | 46.92(18.45,101.50) | 37.35(16.69,73.38) |
| Taiwan (Province of China) | 64.67(35.11,108.56) | 404.35(223.03,678.18) | 1.63(0.88,2.72) | 4.81(2.61,8.07) | 1.52(0.82,2.55) | 3.12(1.73,5.21) | 38.21(20.70,64.41) | 68.57(38.15,113.78) |
| Kingdom of Cambodia | 27.18(9.46,63.81) | 66.02(26.82,142.06) | 2.26(0.78,5.34) | 2.03(0.82,4.40) | 2.59(0.88,6.26) | 2.31(0.93,5.03) | 60.67(20.96,142.76) | 50.76(20.40,110.48) |
| Republic of Indonesia | 259.11(147.33,403.13) | 950.55(473.36,1572.30) | 0.99(0.56,1.53) | 1.49(0.73,2.46) | 1.07(0.60,1.67) | 1.63(0.78,2.70) | 26.27(14.95,40.92) | 37.42(18.46,61.98) |
| Lao People's Democratic Republic | 13.32(6.04,25.29) | 22.29(9.84,41.79) | 2.35(1.06,4.49) | 1.82(0.81,3.41) | 2.69(1.20,5.19) | 2.06(0.92,3.88) | 64.30(28.86,123.20) | 46.80(20.70,87.76) |
| Malaysia | 31.52(15.09,57.44) | 167.30(80.65,305.26) | 1.30(0.62,2.37) | 2.30(1.11,4.23) | 1.47(0.70,2.67) | 2.39(1.16,4.38) | 33.25(16.03,60.48) | 52.82(25.49,97.09) |
| Republic of Maldives | 0.39(0.17,0.74) | 1.28(0.59,2.41) | 1.69(0.74,3.22) | 1.62(0.75,3.03) | 1.99(0.87,3.82) | 1.78(0.84,3.32) | 43.75(19.15,83.57) | 34.95(16.03,65.46) |
| Republic of the Union of Myanmar | 52.71(16.39,118.10) | 122.95(42.41,275.44) | 0.84(0.26,1.87) | 0.94(0.33,2.12) | 0.95(0.30,2.15) | 1.04(0.35,2.31) | 22.68(7.05,51.65) | 23.49(8.15,52.48) |
| Republic of the Philippines | 118.45(72.00,186.30) | 383.89(268.06,522.90) | 1.53(0.93,2.42) | 1.75(1.22,2.37) | 1.73(1.05,2.77) | 1.93(1.36,2.62) | 40.08(24.29,63.23) | 44.43(30.93,60.55) |
| Democratic Socialist Republic of Sri Lanka | 16.18(8.13,28.70) | 36.95(16.64,70.11) | 0.57(0.29,1.02) | 0.53(0.24,1.00) | 0.68(0.34,1.20) | 0.52(0.23,0.98) | 14.47(7.26,25.68) | 11.35(5.01,21.58) |
| Kingdom of Thailand | 376.57(185.37,669.74) | 1155.96(566.17,2092.64) | 4.05(1.99,7.19) | 4.15(2.02,7.52) | 4.46(2.19,7.88) | 3.95(1.96,7.12) | 102.91(50.67,181.32) | 87.93(42.49,160.03) |
| Democratic Republic of Timor-Leste | 0.70(0.29,1.34) | 2.19(0.95,4.35) | 0.99(0.42,1.89) | 0.93(0.40,1.85) | 1.14(0.49,2.17) | 1.05(0.46,2.07) | 26.06(11.14,49.66) | 23.71(10.19,46.66) |
| Socialist Republic of Viet Nam | 363.84(172.04,671.55) | 960.45(434.82,1805.26) | 3.35(1.58,6.20) | 3.81(1.74,7.15) | 3.63(1.71,6.71) | 3.65(1.68,6.74) | 87.30(40.89,162.51) | 86.49(39.36,161.60) |
| Republic of Fiji | 1.17(0.51,2.31) | 3.83(1.78,7.16) | 1.24(0.54,2.45) | 1.83(0.86,3.42) | 1.37(0.60,2.73) | 2.01(0.95,3.69) | 33.11(14.40,65.35) | 47.14(21.89,87.21) |
| Republic of Kiribati | 0.20(0.10,0.39) | 0.43(0.19,0.79) | 2.03(0.95,3.87) | 2.17(0.97,4.05) | 2.26(1.06,4.28) | 2.43(1.09,4.56) | 55.99(26.29,106.49) | 58.28(25.98,108.99) |
| Republic of the Marshall Islands | 0.04(0.02,0.09) | 0.12(0.05,0.23) | 0.92(0.36,2.00) | 1.24(0.53,2.45) | 1.04(0.40,2.28) | 1.38(0.58,2.72) | 24.75(9.69,53.73) | 32.99(13.84,65.59) |
| Federated States of Micronesia | 0.19(0.09,0.36) | 0.38(0.17,0.74) | 1.44(0.65,2.74) | 1.86(0.81,3.65) | 1.56(0.71,2.95) | 2.01(0.90,3.92) | 39.59(17.72,75.36) | 49.13(21.55,95.94) |
| Independent State of Papua New Guinea | 4.32(1.38,12.26) | 9.19(2.88,25.31) | 0.92(0.29,2.61) | 0.69(0.22,1.90) | 1.04(0.34,3.00) | 0.76(0.24,2.10) | 24.61(7.87,70.61) | 18.11(5.70,50.22) |
| Independent State of Samoa | 0.39(0.18,0.72) | 0.68(0.32,1.23) | 1.69(0.79,3.13) | 1.76(0.83,3.20) | 1.93(0.92,3.58) | 1.91(0.91,3.45) | 43.86(20.60,82.22) | 43.77(20.48,80.14) |
| Solomon Islands | 0.54(0.15,1.46) | 1.24(0.51,2.54) | 1.43(0.41,3.86) | 1.32(0.55,2.69) | 1.57(0.46,4.27) | 1.38(0.57,2.82) | 39.54(11.26,107.34) | 35.40(14.50,72.47) |
| Kingdom of Tonga | 0.98(0.43,1.90) | 1.73(0.82,3.19) | 6.54(2.85,12.63) | 8.07(3.79,14.95) | 7.05(3.07,13.63) | 8.42(3.99,15.38) | 171.23(74.61,331.46) | 201.69(93.97,372.90) |
| Republic of Vanuatu | 0.20(0.08,0.46) | 0.62(0.27,1.26) | 1.22(0.48,2.85) | 1.35(0.58,2.73) | 1.43(0.57,3.38) | 1.56(0.67,3.15) | 32.71(12.99,76.83) | 35.42(15.11,71.30) |
| Republic of Armenia | 14.91(8.03,24.69) | 25.24(13.80,42.12) | 2.01(1.08,3.31) | 2.02(1.10,3.35) | 2.42(1.31,3.99) | 2.34(1.28,3.91) | 50.48(27.37,83.12) | 48.83(26.70,81.30) |
| Republic of Azerbaijan | 21.67(9.29,42.54) | 66.59(28.39,130.78) | 1.61(0.69,3.16) | 2.41(1.04,4.74) | 1.87(0.80,3.73) | 2.80(1.20,5.55) | 41.77(17.97,82.05) | 61.23(26.23,120.51) |
| Georgia | 21.13(11.41,35.41) | 18.72(10.28,31.12) | 1.19(0.64,2.00) | 1.13(0.62,1.87) | 1.32(0.72,2.20) | 1.24(0.68,2.06) | 30.80(16.70,51.35) | 28.43(15.56,47.18) |
| Republic of Kazakhstan | 80.03(44.35,132.88) | 72.07(39.51,118.60) | 2.33(1.29,3.87) | 1.51(0.83,2.47) | 2.51(1.40,4.20) | 1.73(0.96,2.86) | 61.45(34.12,102.31) | 37.44(20.57,61.67) |
| Kyrgyz Republic | 11.24(5.91,18.94) | 11.42(5.99,19.41) | 1.39(0.73,2.34) | 0.91(0.48,1.55) | 1.53(0.81,2.59) | 1.01(0.54,1.72) | 36.51(19.26,61.60) | 22.83(12.01,38.74) |
| Mongolia | 28.76(14.12,52.85) | 102.71(52.34,183.16) | 9.95(4.87,18.28) | 17.17(8.72,30.69) | 11.22(5.52,20.66) | 20.76(10.44,37.35) | 270.83(133.65,498.32) | 439.33(222.79,784.26) |
| Republic of Tajikistan | 7.43(3.11,15.66) | 14.17(6.26,27.30) | 1.00(0.42,2.08) | 0.94(0.42,1.81) | 1.18(0.48,2.59) | 1.12(0.49,2.14) | 25.93(11.11,52.98) | 24.33(10.72,46.36) |
| Turkmenistan | 5.73(3.11,9.67) | 17.88(9.50,30.21) | 1.11(0.61,1.88) | 1.63(0.87,2.76) | 1.25(0.68,2.10) | 1.78(0.95,3.02) | 29.41(15.97,49.46) | 42.54(22.54,72.23) |
| Republic of Uzbekistan | 22.64(11.69,40.38) | 86.19(45.03,149.40) | 0.74(0.38,1.31) | 1.19(0.62,2.06) | 0.80(0.41,1.42) | 1.31(0.68,2.24) | 19.43(10.01,34.29) | 31.39(16.26,54.24) |
| Republic of Albania | 19.70(9.91,34.60) | 34.86(16.75,64.29) | 3.56(1.79,6.26) | 2.71(1.30,4.99) | 4.46(2.24,7.81) | 3.23(1.54,5.99) | 88.29(44.23,155.02) | 62.71(29.66,116.29) |
| Bosnia and Herzegovina | 20.77(11.07,34.77) | 40.58(20.81,69.74) | 1.88(1.00,3.14) | 2.23(1.14,3.85) | 2.18(1.16,3.65) | 2.53(1.28,4.30) | 47.69(25.46,79.95) | 52.21(26.31,89.39) |
| Republic of Bulgaria | 75.58(41.27,126.35) | 40.24(21.25,68.17) | 2.17(1.18,3.63) | 0.99(0.52,1.69) | 2.68(1.46,4.46) | 1.13(0.59,1.91) | 55.11(29.94,91.84) | 24.42(12.72,41.42) |
| Republic of Croatia | 15.44(8.39,25.63) | 32.82(17.78,53.88) | 0.94(0.51,1.56) | 1.40(0.75,2.33) | 1.09(0.60,1.81) | 1.25(0.68,2.06) | 21.37(11.58,35.50) | 24.71(13.18,40.92) |
| Czech Republic | 46.60(26.17,78.13) | 47.42(26.21,79.34) | 1.14(0.64,1.92) | 0.77(0.42,1.29) | 1.35(0.76,2.26) | 0.81(0.44,1.36) | 28.72(16.15,48.40) | 16.44(8.91,27.62) |
| Hungary | 33.30(18.18,55.18) | 31.46(16.63,53.10) | 0.77(0.42,1.27) | 0.57(0.30,0.97) | 0.92(0.50,1.53) | 0.61(0.33,1.04) | 19.42(10.61,32.13) | 13.15(6.96,22.36) |
| North Macedonia | 13.78(7.32,23.58) | 25.40(12.97,44.65) | 2.66(1.41,4.55) | 2.84(1.44,5.03) | 3.19(1.69,5.45) | 3.66(1.86,6.39) | 67.46(35.69,115.05) | 67.53(33.99,118.70) |
| Montenegro | 3.04(1.55,5.28) | 6.83(3.55,11.74) | 1.78(0.91,3.09) | 2.51(1.29,4.34) | 2.01(1.03,3.50) | 2.87(1.47,4.93) | 42.53(21.80,73.55) | 56.34(28.81,97.18) |
| Republic of Poland | 24.31(18.27,31.27) | 114.54(84.90,147.88) | 0.19(0.14,0.24) | 0.54(0.40,0.71) | 0.24(0.18,0.31) | 0.63(0.47,0.82) | 4.64(3.48,6.00) | 13.12(9.74,17.06) |
| Romania | 25.47(13.78,43.15) | 98.48(54.35,164.62) | 0.32(0.17,0.54) | 0.95(0.52,1.60) | 0.38(0.21,0.64) | 1.03(0.57,1.73) | 8.16(4.40,13.79) | 22.66(12.43,38.24) |
| Republic of Serbia | 37.12(18.47,67.16) | 65.11(32.34,115.61) | 1.20(0.59,2.19) | 1.39(0.69,2.47) | 1.43(0.71,2.63) | 1.52(0.75,2.71) | 30.12(14.97,55.06) | 31.96(15.80,57.15) |
| Slovak Republic | 22.98(11.80,40.41) | 30.83(14.67,55.68) | 1.34(0.69,2.36) | 1.16(0.55,2.10) | 1.54(0.79,2.73) | 1.26(0.60,2.30) | 33.71(17.24,59.39) | 26.47(12.40,48.24) |
| Republic of Slovenia | 8.08(4.44,13.40) | 21.56(11.75,36.68) | 1.16(0.63,1.92) | 1.86(1.00,3.13) | 1.29(0.71,2.13) | 1.76(0.96,2.98) | 27.99(15.39,46.20) | 34.94(18.81,58.90) |
| Republic of Belarus | 22.20(12.07,37.36) | 34.02(17.83,58.43) | 0.61(0.33,1.02) | 0.78(0.41,1.35) | 0.67(0.37,1.13) | 0.82(0.43,1.42) | 15.32(8.33,25.60) | 18.53(9.55,32.19) |
| Republic of Estonia | 4.69(2.52,7.86) | 9.42(5.12,15.72) | 0.80(0.43,1.34) | 1.26(0.68,2.10) | 0.90(0.48,1.50) | 1.31(0.71,2.19) | 20.26(10.91,33.79) | 27.67(14.98,46.18) |
| Republic of Latvia | 6.85(3.67,11.35) | 10.42(5.65,17.48) | 0.68(0.36,1.12) | 0.93(0.50,1.56) | 0.75(0.40,1.25) | 1.00(0.54,1.69) | 17.29(9.24,28.65) | 21.79(11.72,36.70) |
| Republic of Lithuania | 7.32(3.97,12.14) | 16.19(8.83,27.40) | 0.59(0.32,0.97) | 1.11(0.60,1.88) | 0.63(0.34,1.04) | 1.03(0.56,1.73) | 13.81(7.47,22.69) | 22.86(12.44,38.42) |
| Republic of Moldova | 7.39(4.14,12.14) | 12.92(7.15,21.32) | 0.61(0.34,1.00) | 0.77(0.43,1.28) | 0.69(0.39,1.14) | 0.85(0.47,1.41) | 15.81(8.86,25.95) | 19.02(10.41,31.57) |
| Russian Federation | 200.37(150.27,259.78) | 540.77(398.34,705.74) | 0.40(0.30,0.51) | 0.81(0.59,1.05) | 0.44(0.33,0.57) | 0.90(0.66,1.17) | 10.28(7.69,13.34) | 19.51(14.42,25.51) |
| Ukraine | 93.45(64.32,133.40) | 82.15(51.64,119.71) | 0.47(0.32,0.67) | 0.40(0.25,0.59) | 0.49(0.34,0.70) | 0.40(0.25,0.58) | 11.78(8.12,16.74) | 9.61(5.89,14.07) |
| Brunei Darussalam | 0.72(0.33,1.35) | 2.06(1.01,3.80) | 2.88(1.33,5.38) | 2.51(1.22,4.63) | 3.16(1.47,5.89) | 2.55(1.23,4.71) | 70.25(32.63,131.42) | 53.23(25.81,98.88) |
| Japan | 982.08(740.76,1255.74) | 1821.87(1255.07,2441.72) | 3.22(2.40,4.17) | 3.01(2.08,4.12) | 1.74(1.31,2.23) | 1.18(0.83,1.57) | 41.19(31.07,52.52) | 21.40(15.34,28.31) |
| Republic of Korea | 552.45(276.25,982.92) | 1276.11(646.43,2283.13) | 7.48(3.72,13.30) | 8.50(4.21,15.26) | 7.90(3.89,14.13) | 3.75(1.89,6.64) | 171.99(85.62,306.32) | 73.98(37.41,130.76) |
| Republic of Singapore | 7.59(3.97,13.21) | 34.10(17.07,59.23) | 1.47(0.77,2.55) | 2.60(1.29,4.65) | 1.37(0.71,2.39) | 1.17(0.59,2.01) | 29.64(15.42,51.42) | 23.04(11.72,39.75) |
| Australia | 26.44(14.32,43.76) | 264.37(146.35,433.16) | 0.53(0.29,0.88) | 2.93(1.61,4.83) | 0.52(0.28,0.85) | 1.99(1.10,3.25) | 11.60(6.27,19.18) | 43.10(23.85,70.30) |
| New Zealand | 7.02(4.91,9.49) | 46.10(32.19,62.81) | 0.82(0.57,1.12) | 3.34(2.25,4.69) | 0.58(0.41,0.79) | 1.49(1.05,2.01) | 13.44(9.46,18.18) | 33.14(23.44,44.58) |
| Principality of Andorra | 0.42(0.19,0.81) | 1.36(0.61,2.57) | 2.90(1.31,5.61) | 3.70(1.65,6.96) | 2.80(1.26,5.38) | 3.08(1.40,5.76) | 59.53(27.03,113.83) | 62.89(28.24,118.29) |
| Republic of Austria | 17.40(9.50,29.83) | 62.56(33.65,105.35) | 0.58(0.31,0.99) | 1.81(0.95,3.10) | 0.53(0.29,0.91) | 1.08(0.58,1.80) | 11.31(6.15,19.25) | 22.30(11.99,37.28) |
| Kingdom of Belgium | 26.71(14.07,45.52) | 62.85(32.64,108.08) | 0.62(0.32,1.05) | 1.16(0.61,1.98) | 0.65(0.34,1.12) | 0.94(0.49,1.61) | 12.79(6.74,21.75) | 19.32(10.07,32.73) |
| Republic of Cyprus | 1.48(0.70,2.77) | 4.58(2.19,8.48) | 0.69(0.32,1.31) | 0.96(0.45,1.79) | 0.87(0.40,1.69) | 0.86(0.41,1.62) | 15.93(7.45,30.16) | 15.92(7.52,29.68) |
| Kingdom of Denmark | 8.26(4.50,14.09) | 26.31(13.79,43.80) | 0.55(0.29,0.95) | 1.07(0.55,1.79) | 0.31(0.17,0.53) | 0.70(0.37,1.16) | 6.80(3.67,11.44) | 14.21(7.55,23.60) |
| Republic of Finland | 16.50(8.64,28.04) | 52.85(27.46,89.62) | 1.10(0.58,1.87) | 2.57(1.30,4.46) | 0.75(0.39,1.27) | 1.11(0.59,1.87) | 15.44(8.13,26.04) | 21.59(11.49,36.17) |
| French Republic | 217.42(114.87,369.41) | 663.49(349.67,1132.63) | 1.01(0.53,1.71) | 2.48(1.30,4.26) | 1.00(0.53,1.70) | 1.53(0.81,2.60) | 21.90(11.53,37.02) | 32.22(17.04,54.13) |
| Federal Republic of Germany | 236.36(128.16,395.37) | 722.09(387.84,1217.61) | 0.74(0.40,1.24) | 2.21(1.17,3.77) | 0.69(0.37,1.15) | 1.16(0.63,1.96) | 13.92(7.53,23.28) | 24.00(12.98,40.34) |
| Hellenic Republic | 20.64(11.53,33.91) | 74.20(40.42,121.16) | 0.51(0.28,0.84) | 1.21(0.66,2.00) | 0.53(0.30,0.87) | 1.07(0.59,1.76) | 9.81(5.46,16.09) | 21.40(11.73,35.14) |
| Republic of Iceland | 0.50(0.27,0.86) | 2.02(1.08,3.38) | 0.73(0.38,1.24) | 1.63(0.87,2.75) | 0.65(0.34,1.12) | 1.17(0.63,1.96) | 14.02(7.37,23.93) | 23.34(12.58,38.76) |
| Ireland | 5.24(2.84,8.95) | 21.97(11.73,36.82) | 0.47(0.25,0.79) | 1.28(0.67,2.17) | 0.49(0.27,0.85) | 0.96(0.51,1.61) | 10.02(5.40,17.05) | 19.02(10.14,31.75) |
| State of Israel | 8.66(4.55,14.58) | 25.78(13.98,43.16) | 0.63(0.33,1.07) | 0.87(0.47,1.44) | 0.69(0.36,1.17) | 0.76(0.41,1.27) | 14.04(7.38,23.65) | 15.44(8.40,25.62) |
| Republic of Italy | 256.49(185.77,336.01) | 331.17(232.05,444.38) | 1.13(0.81,1.49) | 1.20(0.84,1.64) | 1.03(0.75,1.35) | 0.72(0.51,0.96) | 22.44(16.30,29.47) | 14.65(10.44,19.49) |
| Grand Duchy of Luxembourg | 1.08(0.59,1.81) | 3.06(1.66,5.14) | 0.72(0.39,1.20) | 1.26(0.67,2.13) | 0.76(0.42,1.27) | 1.02(0.55,1.72) | 15.69(8.56,26.09) | 20.02(10.84,33.39) |
| Republic of Malta | 0.54(0.29,0.92) | 2.02(1.07,3.50) | 0.47(0.25,0.80) | 0.87(0.46,1.50) | 0.50(0.26,0.85) | 0.72(0.38,1.23) | 10.21(5.39,17.39) | 14.82(7.83,25.34) |
| Kingdom of the Netherlands | 18.77(10.14,30.72) | 77.59(42.06,127.08) | 0.32(0.17,0.53) | 0.78(0.42,1.27) | 0.38(0.20,0.62) | 0.85(0.46,1.39) | 7.87(4.25,12.92) | 17.52(9.57,28.70) |
| Kingdom of Norway | 7.05(5.15,9.17) | 26.31(19.08,34.51) | 0.41(0.30,0.54) | 1.30(0.93,1.74) | 0.38(0.28,0.49) | 0.87(0.64,1.15) | 8.09(5.94,10.53) | 18.12(13.28,23.73) |
| Portuguese Republic | 15.56(8.19,26.14) | 72.90(38.17,123.72) | 0.39(0.20,0.65) | 1.20(0.62,2.04) | 0.46(0.24,0.77) | 1.12(0.59,1.91) | 9.36(4.94,15.66) | 24.92(13.11,42.29) |
| Kingdom of Spain | 103.18(54.69,175.03) | 314.49(162.51,548.21) | 0.71(0.37,1.20) | 1.59(0.81,2.76) | 0.72(0.38,1.22) | 1.08(0.56,1.88) | 15.29(8.11,25.79) | 23.09(11.92,39.68) |
| Kingdom of Sweden | 27.90(19.77,37.74) | 47.41(32.47,64.57) | 0.64(0.45,0.87) | 0.84(0.57,1.15) | 0.72(0.51,0.98) | 0.83(0.57,1.14) | 15.30(10.82,20.74) | 17.18(11.72,23.64) |
| Swiss Confederation | 22.91(12.25,38.73) | 51.11(26.68,88.21) | 0.96(0.51,1.62) | 1.51(0.77,2.62) | 0.78(0.42,1.32) | 0.92(0.48,1.58) | 16.96(9.06,28.54) | 18.55(9.78,31.41) |
| United Kingdom of Great Britain and Northern Ireland | 120.87(87.64,161.20) | 617.54(440.55,824.11) | 0.56(0.41,0.75) | 2.51(1.79,3.35) | 0.47(0.34,0.63) | 1.48(1.06,1.99) | 9.88(7.13,13.21) | 29.43(21.15,39.29) |
| Argentine Republic | 12.63(6.67,21.23) | 55.52(29.96,91.56) | 0.14(0.07,0.23) | 0.36(0.19,0.59) | 0.16(0.08,0.27) | 0.39(0.21,0.64) | 3.47(1.83,5.82) | 8.56(4.63,14.12) |
| Republic of Chile | 13.11(7.13,21.94) | 89.02(48.13,145.24) | 0.47(0.26,0.79) | 1.30(0.70,2.12) | 0.54(0.30,0.90) | 1.32(0.71,2.15) | 11.68(6.37,19.55) | 27.49(14.90,44.62) |
| Eastern Republic of Uruguay | 2.83(1.54,4.83) | 11.68(6.28,19.42) | 0.26(0.14,0.44) | 0.80(0.43,1.33) | 0.28(0.15,0.48) | 0.82(0.44,1.35) | 6.28(3.41,10.73) | 18.27(9.77,30.40) |
| Canada | 79.98(46.50,127.76) | 509.46(285.52,829.05) | 1.04(0.60,1.67) | 3.35(1.83,5.49) | 0.91(0.52,1.44) | 2.35(1.32,3.80) | 19.02(10.94,30.37) | 46.96(26.29,76.39) |
| United States of America | 680.87(514.90,860.43) | 3296.95(2459.80,4227.32) | 0.89(0.67,1.12) | 2.83(2.11,3.63) | 0.71(0.53,0.89) | 1.66(1.23,2.12) | 14.81(11.25,18.74) | 35.79(26.83,45.78) |
| Antigua and Barbuda | 0.13(0.07,0.21) | 0.26(0.15,0.42) | 0.81(0.44,1.34) | 0.87(0.49,1.42) | 0.93(0.51,1.54) | 1.00(0.56,1.63) | 20.16(10.99,33.25) | 20.79(11.66,33.77) |
| Commonwealth of the Bahamas | 0.41(0.23,0.68) | 1.14(0.63,1.86) | 1.00(0.56,1.65) | 1.03(0.57,1.68) | 1.12(0.62,1.84) | 1.16(0.64,1.90) | 25.58(14.20,41.98) | 25.49(13.93,41.85) |
| Barbados | 0.63(0.36,1.02) | 1.44(0.78,2.41) | 0.72(0.40,1.18) | 0.96(0.51,1.61) | 0.85(0.48,1.39) | 1.11(0.59,1.84) | 17.82(9.89,29.06) | 22.41(11.85,37.51) |
| Belize | 0.15(0.08,0.24) | 0.67(0.37,1.09) | 0.56(0.31,0.93) | 0.85(0.47,1.38) | 0.66(0.36,1.08) | 0.96(0.52,1.54) | 14.11(7.74,23.14) | 21.21(11.51,34.33) |
| Republic of Cuba | 19.90(10.86,32.88) | 34.76(18.97,57.95) | 0.68(0.37,1.13) | 0.65(0.35,1.08) | 0.79(0.43,1.31) | 0.68(0.37,1.13) | 16.53(8.96,27.29) | 14.72(7.99,24.60) |
| Commonwealth of Dominica | 0.14(0.06,0.26) | 0.32(0.16,0.57) | 0.77(0.36,1.47) | 1.31(0.64,2.32) | 0.94(0.43,1.81) | 1.63(0.80,2.85) | 19.29(9.01,36.62) | 32.16(15.65,56.94) |
| Dominican Republic | 3.41(1.61,6.33) | 16.23(7.63,30.60) | 0.35(0.16,0.65) | 0.59(0.28,1.11) | 0.41(0.19,0.76) | 0.67(0.31,1.26) | 8.79(4.14,16.20) | 14.79(6.88,27.79) |
| Grenada | 0.13(0.07,0.22) | 0.39(0.21,0.64) | 0.59(0.31,1.01) | 1.21(0.66,2.01) | 0.69(0.37,1.20) | 1.48(0.82,2.45) | 15.09(7.89,25.85) | 29.77(16.26,49.38) |
| Republic of Guyana | 0.70(0.38,1.17) | 1.30(0.68,2.21) | 0.68(0.37,1.14) | 0.73(0.39,1.25) | 0.80(0.44,1.33) | 0.84(0.44,1.41) | 18.12(9.93,30.18) | 19.21(10.04,32.31) |
| Republic of Haiti | 3.73(1.45,8.46) | 7.38(2.67,16.98) | 0.43(0.17,0.99) | 0.39(0.14,0.91) | 0.53(0.20,1.23) | 0.48(0.17,1.13) | 11.57(4.45,26.20) | 10.28(3.69,24.06) |
| Jamaica | 1.83(0.97,3.04) | 5.44(2.83,9.25) | 0.35(0.19,0.59) | 0.63(0.33,1.07) | 0.41(0.22,0.68) | 0.71(0.37,1.20) | 8.67(4.58,14.44) | 15.29(7.84,25.98) |
| Saint Lucia | 0.14(0.08,0.24) | 0.32(0.18,0.53) | 0.59(0.32,0.97) | 0.48(0.26,0.79) | 0.72(0.39,1.18) | 0.54(0.29,0.89) | 14.98(8.24,24.55) | 11.81(6.32,19.48) |
| Saint Vincent and the Grenadines | 0.18(0.10,0.30) | 0.33(0.18,0.54) | 0.87(0.47,1.45) | 0.81(0.44,1.34) | 1.04(0.57,1.74) | 0.93(0.51,1.53) | 22.02(11.94,37.05) | 20.23(10.98,33.25) |
| Republic of Suriname | 0.42(0.20,0.76) | 1.55(0.73,2.82) | 0.61(0.30,1.10) | 0.88(0.41,1.60) | 0.71(0.34,1.27) | 0.98(0.47,1.77) | 15.58(7.56,28.10) | 22.19(10.42,40.29) |
| Republic of Trinidad and Tobago | 1.75(0.99,2.83) | 4.73(2.55,7.84) | 0.75(0.42,1.22) | 0.88(0.47,1.46) | 0.90(0.51,1.46) | 0.97(0.51,1.60) | 19.27(10.77,31.06) | 21.89(11.49,36.52) |
| Plurinational State of Bolivia | 8.90(3.86,17.53) | 34.93(15.06,69.19) | 1.03(0.44,2.02) | 1.38(0.60,2.73) | 1.27(0.56,2.49) | 1.73(0.75,3.43) | 26.58(11.53,51.95) | 34.43(14.72,68.92) |
| Republic of Ecuador | 19.78(10.90,32.04) | 61.81(33.64,101.61) | 1.36(0.75,2.21) | 1.30(0.70,2.15) | 1.66(0.92,2.70) | 1.62(0.89,2.65) | 34.10(18.72,55.32) | 31.09(16.78,51.27) |
| Republic of Peru | 17.98(8.61,33.09) | 85.46(41.81,153.59) | 0.55(0.27,1.02) | 0.92(0.45,1.65) | 0.67(0.32,1.22) | 1.07(0.52,1.90) | 13.91(6.73,25.60) | 21.35(10.37,38.24) |
| Republic of Colombia | 38.43(21.02,63.76) | 140.71(75.97,232.84) | 0.82(0.45,1.37) | 0.91(0.49,1.49) | 0.97(0.53,1.62) | 1.05(0.56,1.72) | 20.71(11.32,34.41) | 20.68(11.10,33.95) |
| Republic of Costa Rica | 6.30(3.39,10.50) | 33.81(17.96,56.11) | 1.34(0.72,2.22) | 2.27(1.20,3.75) | 1.53(0.82,2.56) | 2.48(1.31,4.12) | 32.29(17.46,53.50) | 51.06(26.96,84.29) |
| Republic of El Salvador | 4.43(2.30,7.57) | 10.73(5.48,18.65) | 0.55(0.29,0.94) | 0.63(0.33,1.10) | 0.63(0.33,1.07) | 0.69(0.35,1.20) | 14.12(7.37,23.95) | 15.43(7.89,26.67) |
| Republic of Guatemala | 16.48(9.31,27.13) | 50.04(27.30,82.55) | 1.80(1.01,2.96) | 1.65(0.90,2.73) | 2.19(1.24,3.62) | 1.96(1.06,3.22) | 46.87(26.48,77.08) | 41.44(22.36,67.72) |
| Republic of Honduras | 3.07(1.17,7.04) | 22.21(10.18,42.38) | 0.57(0.22,1.30) | 1.28(0.59,2.46) | 0.67(0.25,1.58) | 1.54(0.69,2.95) | 14.57(5.63,33.03) | 32.63(14.72,62.13) |
| United Mexican States | 57.62(43.18,74.12) | 337.13(246.12,442.23) | 0.51(0.38,0.65) | 0.97(0.70,1.27) | 0.60(0.45,0.78) | 1.11(0.81,1.46) | 12.81(9.56,16.51) | 23.54(17.10,30.91) |
| Republic of Nicaragua | 3.25(1.59,5.90) | 13.44(6.45,23.72) | 0.81(0.40,1.47) | 1.04(0.50,1.84) | 0.93(0.46,1.68) | 1.16(0.56,2.05) | 20.48(10.10,36.94) | 25.34(12.10,44.76) |
| Republic of Panama | 4.09(2.22,6.72) | 13.57(7.21,22.81) | 1.01(0.55,1.65) | 1.11(0.59,1.86) | 1.18(0.64,1.95) | 1.24(0.66,2.08) | 24.61(13.38,40.25) | 25.59(13.59,42.90) |
| Bolivarian Republic of Venezuela | 39.50(22.10,64.52) | 70.72(36.94,118.49) | 1.54(0.86,2.52) | 0.86(0.45,1.43) | 1.76(0.98,2.87) | 0.98(0.51,1.65) | 39.04(21.80,63.38) | 20.81(10.70,34.72) |
| Federative Republic of Brazil | 92.22(69.26,119.22) | 356.81(262.98,463.15) | 0.39(0.29,0.50) | 0.52(0.38,0.67) | 0.45(0.34,0.59) | 0.58(0.42,0.75) | 9.94(7.46,12.86) | 12.63(9.33,16.35) |
| Republic of Paraguay | 2.55(1.25,4.66) | 12.22(5.71,22.25) | 0.42(0.21,0.77) | 0.78(0.36,1.41) | 0.50(0.24,0.90) | 0.88(0.41,1.61) | 10.61(5.24,19.29) | 19.02(8.93,34.44) |
| People's Democratic Republic of Algeria | 12.69(6.18,23.14) | 69.86(34.36,125.28) | 0.39(0.19,0.70) | 0.75(0.37,1.35) | 0.45(0.22,0.81) | 0.85(0.41,1.52) | 9.79(4.76,17.82) | 17.80(8.66,31.82) |
| Kingdom of Bahrain | 1.18(0.59,2.13) | 4.75(2.29,8.84) | 2.89(1.44,5.19) | 2.54(1.25,4.68) | 3.62(1.81,6.53) | 2.98(1.48,5.42) | 73.27(36.68,131.93) | 56.00(27.57,102.89) |
| Arab Republic of Egypt | 264.14(105.79,580.21) | 1276.23(636.72,2192.09) | 3.74(1.49,8.30) | 7.65(3.83,13.19) | 4.39(1.72,9.93) | 8.50(4.23,14.78) | 98.18(39.20,216.74) | 192.93(95.50,332.16) |
| Islamic Republic of Iran | 49.50(33.76,71.66) | 292.89(212.11,387.82) | 0.78(0.53,1.13) | 1.46(1.06,1.94) | 0.98(0.67,1.43) | 1.72(1.25,2.27) | 19.46(13.30,28.27) | 32.86(23.84,43.56) |
| Republic of Iraq | 27.96(13.20,51.28) | 119.77(58.17,214.80) | 1.34(0.63,2.45) | 1.98(0.96,3.56) | 1.48(0.70,2.72) | 2.25(1.09,3.99) | 34.73(16.44,63.13) | 47.85(22.91,84.88) |
| Hashemite Kingdom of Jordan | 2.38(1.07,4.71) | 13.99(6.80,25.09) | 0.71(0.32,1.40) | 0.76(0.37,1.37) | 0.81(0.36,1.61) | 0.83(0.40,1.47) | 17.92(8.03,35.40) | 17.39(8.39,30.77) |
| State of Kuwait | 2.27(1.23,3.76) | 4.55(2.44,7.52) | 1.62(0.88,2.67) | 0.74(0.40,1.22) | 1.70(0.92,2.79) | 0.76(0.41,1.23) | 38.52(20.86,63.22) | 14.61(7.88,23.94) |
| Lebanese Republic | 4.62(2.21,8.54) | 12.76(6.13,23.36) | 0.79(0.38,1.46) | 0.84(0.40,1.54) | 0.90(0.43,1.68) | 0.82(0.39,1.50) | 19.91(9.46,36.91) | 18.47(8.90,33.89) |
| State of Libya | 8.87(3.98,16.99) | 42.66(20.57,79.89) | 1.81(0.82,3.46) | 3.23(1.56,6.06) | 2.01(0.91,3.90) | 3.49(1.68,6.47) | 45.47(20.53,88.19) | 77.66(37.28,142.97) |
| Kingdom of Morocco | 4.27(1.97,7.94) | 18.84(8.81,34.17) | 0.11(0.05,0.21) | 0.20(0.09,0.37) | 0.12(0.06,0.23) | 0.22(0.10,0.40) | 2.89(1.34,5.40) | 5.06(2.34,9.22) |
| Palestine | 5.17(2.35,9.77) | 13.94(7.01,24.66) | 2.19(0.99,4.15) | 2.20(1.11,3.90) | 2.69(1.23,5.09) | 2.58(1.30,4.57) | 55.52(25.37,105.75) | 52.41(26.34,92.95) |
| Sultanate of Oman | 1.43(0.61,2.89) | 8.05(3.86,14.89) | 0.83(0.36,1.68) | 1.72(0.83,3.15) | 0.91(0.39,1.83) | 1.78(0.86,3.24) | 21.63(9.22,43.84) | 39.49(19.22,72.21) |
| State of Qatar | 1.02(0.49,1.89) | 14.90(7.11,27.52) | 4.48(2.15,8.30) | 9.20(4.54,16.81) | 5.51(2.63,10.15) | 9.67(4.84,17.53) | 111.65(53.41,205.91) | 187.80(93.00,341.80) |
| Kingdom of Saudi Arabia | 34.06(14.87,66.04) | 154.58(75.06,273.60) | 2.30(1.00,4.46) | 3.68(1.82,6.47) | 2.58(1.14,5.03) | 4.08(2.01,7.17) | 59.05(25.96,115.46) | 85.51(41.93,150.44) |
| Syrian Arab Republic | 26.20(11.72,50.90) | 70.83(35.49,124.25) | 1.91(0.86,3.72) | 2.04(1.03,3.57) | 2.26(1.01,4.43) | 2.28(1.16,3.97) | 48.80(21.76,94.56) | 48.10(24.02,84.02) |
| Republic of Tunisia | 4.68(2.14,8.79) | 20.80(9.33,39.75) | 0.35(0.16,0.66) | 0.59(0.26,1.13) | 0.40(0.18,0.76) | 0.62(0.28,1.20) | 8.64(3.96,16.43) | 13.55(6.04,26.06) |
| Republic of Turkey | 73.22(34.91,136.67) | 290.47(145.41,514.40) | 0.79(0.38,1.48) | 1.16(0.58,2.06) | 0.94(0.44,1.77) | 1.28(0.65,2.27) | 20.21(9.67,37.61) | 26.06(13.02,46.21) |
| United Arab Emirates | 3.40(1.58,6.23) | 51.77(24.80,94.63) | 3.56(1.66,6.51) | 6.00(3.00,10.60) | 3.92(1.82,7.10) | 6.82(3.43,11.97) | 90.78(42.28,164.45) | 142.06(70.31,250.97) |
| Republic of Yemen | 8.35(2.51,20.96) | 19.93(7.67,43.98) | 0.65(0.19,1.65) | 0.55(0.21,1.22) | 0.75(0.22,1.96) | 0.64(0.24,1.46) | 17.18(5.15,43.36) | 14.08(5.42,31.23) |
| Islamic Republic of Afghanistan | 29.61(12.12,62.43) | 49.26(20.89,100.11) | 1.50(0.61,3.19) | 1.95(0.83,3.98) | 1.76(0.69,3.91) | 2.24(0.93,4.63) | 40.97(16.58,86.87) | 52.07(21.98,104.27) |
| People's Republic of Bangladesh | 63.73(29.96,119.53) | 239.04(112.28,445.85) | 0.51(0.24,0.95) | 0.64(0.30,1.19) | 0.56(0.27,1.05) | 0.71(0.34,1.32) | 13.58(6.40,25.55) | 16.07(7.54,30.02) |
| Kingdom of Bhutan | 0.59(0.24,1.15) | 2.37(1.00,4.65) | 0.92(0.38,1.79) | 1.45(0.61,2.84) | 1.04(0.43,2.03) | 1.62(0.67,3.17) | 24.59(10.03,48.34) | 36.59(15.42,71.37) |
| Republic of India | 998.38(722.49,1324.87) | 4121.88(3051.49,5329.55) | 0.80(0.58,1.06) | 1.27(0.94,1.64) | 0.89(0.65,1.17) | 1.43(1.06,1.84) | 21.36(15.52,28.35) | 32.32(23.85,41.92) |
| Federal Democratic Republic of Nepal | 11.46(5.21,20.92) | 65.89(29.16,127.99) | 0.46(0.21,0.83) | 1.03(0.46,2.01) | 0.52(0.24,0.95) | 1.17(0.52,2.26) | 12.22(5.55,22.44) | 26.50(11.76,51.57) |
| Islamic Republic of Pakistan | 93.22(59.50,138.33) | 281.63(178.69,427.94) | 0.60(0.39,0.90) | 0.86(0.55,1.31) | 0.69(0.44,1.02) | 0.97(0.62,1.47) | 16.10(10.30,23.87) | 22.75(14.44,34.73) |
| Republic of Angola | 27.10(3.64,83.93) | 61.40(12.62,180.00) | 2.59(0.34,8.07) | 1.99(0.41,5.89) | 2.98(0.38,9.44) | 2.31(0.47,7.00) | 71.34(9.44,222.86) | 52.89(10.80,158.02) |
| Central African Republic | 7.70(2.36,18.84) | 9.65(3.01,25.24) | 2.47(0.74,6.23) | 1.64(0.50,4.41) | 2.90(0.83,7.57) | 1.95(0.57,5.43) | 69.23(20.67,173.33) | 45.18(13.77,121.07) |
| Republic of the Congo | 9.09(2.87,23.01) | 15.46(5.17,39.02) | 3.15(0.97,8.13) | 2.17(0.72,5.50) | 3.71(1.10,9.84) | 2.55(0.83,6.63) | 86.71(26.83,223.07) | 57.52(19.06,146.97) |
| Democratic Republic of the Congo | 47.53(17.40,114.85) | 92.30(30.47,255.55) | 1.14(0.41,2.80) | 0.98(0.32,2.73) | 1.32(0.46,3.34) | 1.17(0.36,3.36) | 30.76(11.05,75.47) | 25.76(8.34,72.46) |
| Republic of Equatorial Guinea | 0.50(0.18,1.17) | 2.61(1.13,5.02) | 0.96(0.35,2.26) | 2.03(0.88,3.91) | 1.14(0.41,2.73) | 2.38(1.03,4.60) | 26.26(9.56,61.40) | 52.19(22.34,100.75) |
| Gabonese Republic | 3.88(1.24,9.75) | 8.95(3.85,17.69) | 2.45(0.78,6.21) | 3.19(1.37,6.31) | 2.80(0.86,7.29) | 3.74(1.60,7.44) | 66.01(20.83,167.11) | 83.20(35.68,164.65) |
| Republic of Burundi | 10.71(4.32,22.22) | 14.55(6.15,29.13) | 1.68(0.67,3.48) | 1.15(0.48,2.30) | 1.95(0.78,4.08) | 1.34(0.56,2.69) | 46.05(18.33,96.13) | 30.27(12.71,60.78) |
| Union of the Comoros | 1.36(0.60,2.61) | 3.29(1.43,6.29) | 2.60(1.14,4.97) | 2.49(1.08,4.75) | 3.15(1.41,6.03) | 2.96(1.29,5.60) | 68.68(30.01,132.63) | 64.12(27.58,123.07) |
| Republic of Djibouti | 0.53(0.24,1.05) | 3.38(1.54,6.48) | 1.54(0.70,3.02) | 2.09(0.96,3.99) | 1.78(0.81,3.49) | 2.49(1.14,4.73) | 40.91(18.65,80.13) | 54.15(24.51,103.75) |
| State of Eritrea | 4.52(2.09,8.79) | 11.86(5.28,23.36) | 1.57(0.71,3.10) | 1.68(0.74,3.31) | 1.92(0.87,3.85) | 2.01(0.88,3.97) | 43.47(20.03,85.48) | 44.54(19.68,87.64) |
| Federal Democratic Republic of Ethiopia | 68.70(43.22,108.40) | 115.89(67.50,202.38) | 1.28(0.80,2.06) | 1.01(0.59,1.78) | 1.54(0.94,2.56) | 1.20(0.69,2.10) | 35.53(22.08,57.25) | 26.16(15.23,45.94) |
| Republic of Kenya | 27.28(17.06,44.31) | 139.82(95.78,199.82) | 1.24(0.77,2.01) | 2.31(1.58,3.30) | 1.41(0.88,2.28) | 2.65(1.83,3.75) | 32.69(20.43,53.25) | 60.28(41.22,86.11) |
| Republic of Madagascar | 16.89(7.90,31.71) | 35.81(16.34,68.55) | 1.24(0.58,2.32) | 1.24(0.56,2.36) | 1.41(0.65,2.67) | 1.45(0.65,2.76) | 33.20(15.46,62.36) | 32.57(14.66,61.62) |
| Republic of Malawi | 14.87(6.93,27.54) | 41.08(19.58,75.80) | 1.43(0.66,2.64) | 2.11(1.01,3.88) | 1.65(0.78,3.04) | 2.44(1.17,4.47) | 38.61(18.01,71.45) | 55.68(26.42,102.27) |
| Republic of Mauritius | 2.76(1.51,4.50) | 1.60(0.90,2.58) | 1.44(0.79,2.35) | 0.33(0.18,0.53) | 1.65(0.91,2.69) | 0.33(0.18,0.53) | 36.04(19.79,59.02) | 7.41(4.11,11.95) |
| Republic of Mozambique | 105.68(48.55,199.01) | 263.02(103.44,562.39) | 6.78(3.11,12.79) | 9.40(3.67,20.06) | 8.64(3.96,16.26) | 12.27(4.75,25.91) | 177.62(81.19,333.86) | 244.91(95.49,519.98) |
| Republic of Rwanda | 17.76(7.96,34.01) | 31.64(13.99,58.96) | 2.31(1.04,4.43) | 1.92(0.85,3.59) | 2.74(1.22,5.22) | 2.30(1.02,4.30) | 63.69(28.04,121.52) | 49.74(22.08,92.82) |
| Republic of Seychelles | 0.43(0.21,0.75) | 0.52(0.25,0.93) | 2.71(1.35,4.79) | 1.68(0.81,3.01) | 3.05(1.53,5.37) | 1.77(0.87,3.14) | 69.46(34.66,122.78) | 39.70(19.01,70.96) |
| Federal Republic of Somalia | 21.72(8.10,46.71) | 51.99(19.68,115.16) | 3.52(1.31,7.56) | 3.44(1.29,7.77) | 4.09(1.51,8.91) | 4.21(1.57,9.60) | 96.99(35.87,211.40) | 92.13(34.48,206.10) |
| United Republic of Tanzania | 67.38(32.68,122.76) | 146.50(68.12,272.62) | 2.28(1.10,4.15) | 2.16(1.00,4.01) | 2.63(1.27,4.80) | 2.52(1.17,4.65) | 60.74(29.28,111.34) | 56.10(26.01,104.65) |
| Republic of Uganda | 46.29(21.95,84.86) | 108.09(48.48,207.10) | 2.62(1.24,4.80) | 2.81(1.26,5.39) | 3.07(1.45,5.64) | 3.30(1.49,6.37) | 70.70(33.22,129.98) | 73.04(32.69,140.47) |
| Republic of Zambia | 27.14(12.21,53.24) | 38.54(13.15,100.82) | 3.52(1.58,6.91) | 2.19(0.76,5.63) | 4.04(1.80,7.97) | 2.72(0.98,6.82) | 95.63(42.91,188.46) | 57.16(19.60,150.34) |
| Republic of Botswana | 2.65(0.96,6.12) | 9.14(3.63,20.62) | 1.85(0.66,4.30) | 2.35(0.95,5.24) | 2.29(0.83,5.34) | 2.85(1.17,6.22) | 48.56(17.64,113.17) | 61.88(24.38,140.81) |
| Kingdom of Lesotho | 4.85(1.45,12.14) | 17.47(5.85,43.24) | 2.08(0.62,5.26) | 5.93(2.01,14.58) | 2.47(0.73,6.23) | 7.15(2.46,17.35) | 54.84(16.19,136.82) | 164.52(54.32,407.57) |
| Republic of Namibia | 1.40(0.58,2.77) | 4.31(2.04,7.92) | 0.82(0.34,1.64) | 1.19(0.56,2.19) | 1.00(0.42,2.00) | 1.43(0.68,2.62) | 21.52(8.92,43.04) | 30.67(14.47,56.72) |
| Republic of South Africa | 80.10(44.15,137.84) | 363.17(261.15,489.53) | 1.45(0.80,2.50) | 2.88(2.07,3.88) | 1.70(0.95,2.93) | 3.45(2.50,4.65) | 37.51(20.68,64.38) | 74.56(53.55,100.68) |
| Kingdom of Eswatini | 2.72(0.97,5.69) | 11.57(3.62,28.40) | 3.61(1.30,7.59) | 7.85(2.51,19.06) | 4.38(1.57,9.17) | 9.49(3.11,22.86) | 95.90(34.07,200.01) | 213.22(66.41,531.48) |
| Republic of Zimbabwe | 42.48(19.38,78.27) | 99.65(43.78,193.85) | 3.94(1.79,7.29) | 5.62(2.47,10.97) | 4.72(2.16,8.77) | 7.08(3.13,13.96) | 102.01(46.66,188.44) | 148.12(64.89,290.36) |
| Republic of Benin | 33.56(13.03,71.08) | 69.43(32.99,128.75) | 6.14(2.39,13.02) | 5.10(2.43,9.47) | 7.18(2.78,15.12) | 6.12(2.92,11.43) | 163.42(63.34,344.25) | 131.40(61.65,248.06) |
| Burkina Faso | 80.03(27.45,196.57) | 142.04(53.45,319.07) | 6.74(2.31,16.55) | 5.74(2.16,12.88) | 7.96(2.72,19.75) | 6.85(2.60,15.64) | 180.31(62.04,446.30) | 150.00(56.26,342.18) |
| Republic of Cameroon | 78.12(35.80,145.13) | 169.30(67.79,333.00) | 6.59(3.03,12.24) | 5.21(2.09,10.23) | 7.78(3.59,14.44) | 6.29(2.57,12.25) | 174.34(79.82,325.57) | 134.71(54.26,264.45) |
| Republic of Cabo Verde | 2.51(1.10,5.12) | 7.24(3.26,13.36) | 3.59(1.58,7.24) | 5.94(2.69,10.92) | 4.40(1.89,9.02) | 7.36(3.32,13.45) | 89.63(39.28,181.23) | 143.38(65.14,263.01) |
| Republic of Chad | 33.37(11.03,81.20) | 61.95(24.72,136.01) | 4.24(1.41,10.34) | 4.06(1.62,8.95) | 4.99(1.63,12.25) | 4.91(1.96,10.88) | 113.28(37.21,275.91) | 106.59(42.48,235.40) |
| Republic of Cote d'Ivoire | 20.07(8.86,39.02) | 42.52(17.26,83.42) | 1.95(0.87,3.77) | 1.49(0.60,2.91) | 2.28(1.04,4.35) | 1.75(0.71,3.46) | 51.97(23.22,100.37) | 38.50(15.70,75.98) |
| Republic of the Gambia | 7.56(3.64,13.76) | 31.05(13.28,59.82) | 8.19(3.94,14.93) | 11.93(5.09,22.96) | 9.36(4.57,16.79) | 13.72(5.88,26.25) | 217.91(105.33,395.31) | 312.96(133.87,599.27) |
| Republic of Ghana | 53.52(22.62,111.35) | 137.29(60.65,256.53) | 3.30(1.40,6.87) | 3.19(1.41,5.96) | 4.04(1.72,8.41) | 3.98(1.77,7.41) | 86.32(36.38,181.35) | 80.96(35.83,150.89) |
| Republic of Guinea | 68.97(33.14,124.64) | 106.62(48.17,196.40) | 7.51(3.62,13.57) | 7.03(3.17,12.96) | 8.74(4.22,15.84) | 8.28(3.74,15.24) | 201.09(97.25,362.67) | 185.53(83.47,340.78) |
| Republic of Guinea-Bissau | 9.21(3.29,19.16) | 11.77(5.59,21.86) | 8.57(3.07,17.92) | 6.45(3.08,11.97) | 10.08(3.60,21.10) | 7.95(3.84,14.82) | 236.58(84.26,493.54) | 171.25(81.79,320.34) |
| Republic of Liberia | 22.64(9.30,44.15) | 37.05(17.59,68.73) | 7.12(2.91,13.95) | 6.87(3.29,12.67) | 8.49(3.48,16.59) | 8.25(3.98,15.06) | 189.78(77.33,373.47) | 177.19(84.71,326.22) |
| Republic of Mali | 77.10(38.78,138.72) | 195.32(92.58,363.09) | 7.16(3.60,12.84) | 8.46(4.02,15.72) | 8.31(4.16,14.89) | 10.00(4.83,18.45) | 192.22(96.48,345.12) | 220.97(105.51,409.42) |
| Islamic Republic of Mauritania | 34.71(8.24,85.07) | 52.84(23.13,100.38) | 12.59(2.96,30.82) | 9.10(3.98,17.32) | 14.68(3.45,36.42) | 10.95(4.78,20.58) | 334.04(78.46,820.24) | 228.97(99.42,432.33) |
| Republic of the Niger | 39.37(13.87,90.57) | 76.02(32.51,156.12) | 5.40(1.90,12.47) | 3.53(1.52,7.23) | 6.31(2.23,14.60) | 4.27(1.85,8.75) | 144.71(51.12,333.19) | 91.92(39.52,190.45) |
| Federal Republic of Nigeria | 246.69(107.80,522.86) | 521.58(324.56,817.62) | 2.02(0.89,4.28) | 2.18(1.37,3.39) | 2.44(1.09,5.18) | 2.63(1.70,4.02) | 53.07(23.05,112.91) | 55.95(35.01,87.32) |
| Democratic Republic of Sao Tome and Principe | 0.16(0.08,0.28) | 0.27(0.11,0.55) | 0.85(0.42,1.54) | 0.94(0.37,1.89) | 1.04(0.52,1.87) | 1.15(0.46,2.29) | 21.94(10.88,39.71) | 23.76(9.33,47.67) |
| Republic of Senegal | 42.71(20.50,78.13) | 96.46(45.04,177.66) | 4.89(2.34,8.94) | 4.65(2.17,8.57) | 5.77(2.79,10.46) | 5.63(2.64,10.27) | 128.99(62.11,236.01) | 119.08(55.53,218.77) |
| Republic of Sierra Leone | 29.03(8.95,68.78) | 35.40(16.25,67.20) | 5.11(1.57,12.12) | 3.52(1.62,6.66) | 6.07(1.88,14.18) | 4.24(1.95,7.97) | 136.05(41.44,320.53) | 91.27(41.85,173.72) |
| Togolese Republic | 9.87(4.81,18.05) | 33.91(15.16,64.68) | 3.05(1.49,5.58) | 3.48(1.56,6.65) | 3.61(1.76,6.62) | 4.25(1.90,8.11) | 80.74(39.27,148.49) | 89.58(40.09,171.32) |
| American Samoa | 0.10(0.05,0.18) | 0.40(0.20,0.72) | 1.72(0.84,3.14) | 3.07(1.50,5.54) | 1.92(0.94,3.47) | 3.30(1.63,5.92) | 44.52(21.65,81.24) | 76.30(37.16,136.65) |
| Bermuda | 0.18(0.10,0.29) | 0.25(0.14,0.41) | 1.01(0.57,1.65) | 0.71(0.38,1.17) | 1.19(0.67,1.93) | 0.68(0.37,1.11) | 24.16(13.62,39.33) | 13.72(7.32,22.60) |
| Cook Islands | 0.12(0.06,0.22) | 0.33(0.16,0.60) | 3.61(1.73,6.58) | 5.01(2.43,9.17) | 4.04(1.94,7.37) | 4.64(2.28,8.37) | 91.92(43.75,168.89) | 107.26(51.91,196.66) |
| Greenland | 0.17(0.09,0.30) | 0.46(0.22,0.84) | 1.98(1.02,3.46) | 2.50(1.20,4.54) | 2.30(1.18,4.02) | 2.87(1.39,5.15) | 50.99(26.02,89.82) | 58.71(28.19,106.10) |
| Guam | 0.21(0.11,0.36) | 1.19(0.61,2.05) | 1.09(0.56,1.87) | 2.22(1.14,3.81) | 1.18(0.61,2.03) | 2.10(1.07,3.62) | 26.89(13.80,46.43) | 52.22(26.67,90.43) |
| Principality of Monaco | 0.21(0.10,0.39) | 0.69(0.33,1.25) | 1.17(0.54,2.17) | 3.09(1.45,5.62) | 1.08(0.49,2.02) | 2.42(1.15,4.36) | 22.54(10.45,41.84) | 50.25(23.90,89.44) |
| Republic of Nauru | 0.02(0.01,0.04) | 0.03(0.01,0.05) | 1.91(0.88,3.53) | 1.77(0.79,3.30) | 2.10(0.97,3.87) | 1.71(0.76,3.20) | 52.77(23.94,98.34) | 48.62(21.21,91.69) |
| Republic of Niue | 0.01(0.00,0.02) | 0.01(0.01,0.02) | 1.36(0.60,2.67) | 2.07(0.92,4.07) | 1.51(0.67,2.95) | 2.19(0.99,4.25) | 35.60(15.62,69.97) | 50.86(22.38,100.91) |
| Northern Mariana Islands | 0.09(0.04,0.16) | 0.43(0.21,0.78) | 2.10(1.00,3.91) | 3.27(1.56,5.89) | 2.30(1.10,4.27) | 3.23(1.55,5.79) | 52.95(25.06,98.44) | 75.29(36.15,135.67) |
| Republic of Palau | 0.05(0.02,0.10) | 0.15(0.06,0.30) | 1.77(0.72,3.72) | 2.37(0.97,4.70) | 1.91(0.78,3.99) | 2.38(0.98,4.70) | 47.66(18.97,101.53) | 60.71(24.61,121.18) |
| Puerto Rico | 9.90(5.39,16.30) | 25.92(13.97,43.63) | 0.98(0.53,1.62) | 1.39(0.74,2.34) | 1.10(0.60,1.81) | 1.36(0.73,2.29) | 23.80(12.82,38.99) | 29.85(15.70,50.17) |
| Saint Kitts and Nevis | 0.13(0.07,0.22) | 0.23(0.12,0.38) | 1.13(0.62,1.87) | 1.21(0.67,2.00) | 1.37(0.76,2.26) | 1.41(0.78,2.31) | 29.35(16.22,48.67) | 29.95(16.32,49.29) |
| Republic of San Marino | 0.05(0.02,0.09) | 0.13(0.06,0.25) | 0.54(0.25,1.01) | 0.80(0.34,1.55) | 0.49(0.23,0.92) | 0.60(0.26,1.17) | 10.09(4.72,18.94) | 12.41(5.34,24.31) |
| Tokelau | 0.00(0.00,0.01) | 0.01(0.00,0.01) | 1.20(0.47,2.64) | 1.75(0.75,3.49) | 1.37(0.54,3.03) | 1.76(0.76,3.47) | 31.61(12.39,70.08) | 43.23(18.37,85.75) |
| Tuvalu | 0.02(0.01,0.05) | 0.05(0.02,0.09) | 1.30(0.55,2.73) | 1.66(0.72,3.24) | 1.50(0.62,3.18) | 1.80(0.79,3.51) | 35.31(14.74,74.07) | 42.54(18.50,82.92) |
| United States Virgin Islands | 0.14(0.06,0.27) | 0.55(0.26,1.02) | 0.64(0.29,1.22) | 1.02(0.47,1.92) | 0.76(0.34,1.46) | 1.12(0.52,2.08) | 15.88(7.00,30.34) | 24.96(11.27,46.82) |
| Republic of South Sudan | 14.20(6.22,27.85) | 30.66(13.90,57.21) | 2.04(0.89,4.01) | 3.01(1.37,5.62) | 2.36(1.03,4.57) | 3.52(1.62,6.63) | 54.71(23.69,107.00) | 79.88(36.40,150.22) |
| Republic of Sudan | 24.13(8.66,55.86) | 75.51(33.90,143.79) | 0.96(0.34,2.25) | 1.49(0.67,2.83) | 1.13(0.39,2.70) | 1.75(0.79,3.32) | 25.39(9.12,58.67) | 37.66(16.85,71.91) |

**Supplementary Table 4.** Global prediction age-standardized incidence, prevalence, mortality rate, and DALYs of NALC from 2022 to 2050.

| **Time** | **Prevalence (95%UI)** | **Incidence (95%UI)** | **Deaths (95%UI)** | **DALYs (95%UI)** |
| --- | --- | --- | --- | --- |
| 2022 | 2.04(2.00,2.09) | 1.70(1.66,1.75) | 1.67(1.63,1.72) | 36.89(35.89,37.88) |
| 2023 | 2.06(2.00,2.13) | 1.71(1.66,1.77) | 1.68(1.62,1.73) | 36.91(35.62,38.20) |
| 2024 | 2.08(2.00,2.15) | 1.72(1.66,1.79) | 1.68(1.61,1.75) | 36.93(35.39,38.47) |
| 2025 | 2.09(2.01,2.18) | 1.73(1.65,1.80) | 1.68(1.60,1.76) | 36.94(35.17,38.71) |
| 2026 | 2.11(2.01,2.21) | 1.74(1.65,1.82) | 1.68(1.59,1.77) | 36.93(34.94,38.92) |
| 2027 | 2.13(2.02,2.24) | 1.74(1.65,1.84) | 1.69(1.58,1.79) | 36.91(34.71,39.11) |
| 2028 | 2.14(2.02,2.27) | 1.75(1.65,1.85) | 1.69(1.58,1.80) | 36.88(34.47,39.29) |
| 2029 | 2.16(2.02,2.29) | 1.76(1.64,1.87) | 1.69(1.57,1.81) | 36.85(34.23,39.46) |
| 2030 | 2.17(2.03,2.32) | 1.76(1.64,1.88) | 1.69(1.56,1.82) | 36.80(33.98,39.62) |
| 2031 | 2.19(2.03,2.35) | 1.77(1.64,1.90) | 1.69(1.55,1.83) | 36.74(33.71,39.78) |
| 2032 | 2.20(2.03,2.37) | 1.77(1.63,1.91) | 1.69(1.54,1.83) | 36.67(33.41,39.92) |
| 2033 | 2.22(2.03,2.40) | 1.78(1.63,1.93) | 1.69(1.54,1.84) | 36.58(33.10,40.07) |
| 2034 | 2.23(2.03,2.43) | 1.78(1.62,1.94) | 1.69(1.53,1.85) | 36.50(32.76,40.23) |
| 2035 | 2.24(2.02,2.46) | 1.78(1.61,1.95) | 1.69(1.51,1.86) | 36.40(32.41,40.39) |
| 2036 | 2.25(2.02,2.49) | 1.79(1.61,1.97) | 1.69(1.50,1.87) | 36.29(32.02,40.55) |
| 2037 | 2.26(2.01,2.52) | 1.79(1.60,1.98) | 1.68(1.49,1.87) | 36.16(31.60,40.72) |
| 2038 | 2.27(2.00,2.55) | 1.79(1.59,2.00) | 1.68(1.48,1.88) | 36.03(31.15,40.91) |
| 2039 | 2.29(1.99,2.58) | 1.79(1.58,2.01) | 1.68(1.46,1.89) | 35.89(30.67,41.11) |
| 2040 | 2.29(1.98,2.61) | 1.80(1.56,2.03) | 1.67(1.45,1.90) | 35.75(30.16,41.34) |
| 2041 | 2.30(1.96,2.65) | 1.80(1.55,2.04) | 1.67(1.43,1.91) | 35.60(29.61,41.58) |
| 2042 | 2.31(1.94,2.69) | 1.80(1.53,2.06) | 1.66(1.41,1.92) | 35.43(29.02,41.84) |
| 2043 | 2.32(1.91,2.73) | 1.80(1.51,2.08) | 1.66(1.39,1.93) | 35.26(28.40,42.13) |
| 2044 | 2.33(1.89,2.77) | 1.80(1.49,2.10) | 1.65(1.37,1.94) | 35.09(27.75,42.44) |
| 2045 | 2.34(1.86,2.81) | 1.79(1.47,2.12) | 1.65(1.35,1.95) | 34.92(27.06,42.78) |
| 2046 | 2.34(1.83,2.86) | 1.79(1.45,2.14) | 1.64(1.33,1.96) | 34.74(26.33,43.14) |
| 2047 | 2.35(1.79,2.90) | 1.79(1.42,2.16) | 1.64(1.30,1.97) | 34.55(25.56,43.53) |
| 2048 | 2.36(1.75,2.96) | 1.79(1.40,2.18) | 1.63(1.28,1.98) | 34.35(24.76,43.95) |
| 2049 | 2.36(1.71,3.01) | 1.79(1.37,2.21) | 1.62(1.25,2.00) | 34.16(23.92,44.40) |
| 2050 | 2.37(1.67,3.06) | 1.78(1.34,2.23) | 1.62(1.22,2.01) | 33.96(23.05,44.87) |
